# A Discriminative PCR Assay Enabling Differentiation of ‘*Candidatus* Phytoplasma solani’ Subgroups 16SrXII-A and -P

**DOI:** 10.3390/plants15182823

**Published:** 2026-09-14

**Authors:** Rafael Toth, Nadine Fluck, Johannes Ritz, Mark Varrelmann, Michael Kube

**Affiliations:** 1 Department of Integrative Infection Biology Crops-Livestock, University of Hohenheim, 70599 Stuttgart, Germany; nadine.fluck@uni-hohenheim.de; 2 Bioland Beratungsdienst GmbH, 73728 Esslingen am Neckar, Germany; johannes.ritz@bioland.de; 3 Department of Phytopathology, Institute of Sugar Beet Research (IfZ), 37079 Göttingen, Germany; varrelmann@ifz-goettingen.de

**Keywords:** stolbur, SBR, *Beta vulgaris* subsp. *vulgaris*, sugar beet, red beet, *P. leporinus*, *secY*, PCR

## Abstract

Phytoplasmosis associated with the ‘*Candidatus* Phytoplasma solani’ subgroups 16SrXII-A and 16SrXII-P (Mollicutes) poses a threat to European sugar beet cultivation, alongside the “basses richesses” syndrome caused by ‘*Ca*. Arsenophonus phytopathogenicus’ (Gammaproteobacteria). Both subgroups co-occur in sugar beet and have been detected in *Pentastiridius leporinus*, a polyphagous planthopper associated with SBR-affected fields. Despite ongoing monitoring of disease spread and pathogen characterization, approaches for direct subgroup differentiation remain limited. Consequently, discriminative conventional PCR assays targeting *secY* were developed, with detection limits estimated using synthetic gene fragments. Assay performance was assessed using 900 DNA templates from sugar beet from Germany and neighboring countries, 50 German red beet samples, and 320 planthoppers. Distinct amplicon sizes of 549 bp and 857 bp enabled reliable detection and differentiation of 16SrXII-A and 16SrXII-P, respectively. Detection rates were broadly concordant with those obtained using an established phytoplasma detection approach. Estimated detection limits were 100 target copies for 16SrXII-A and 1000 for 16SrXII-P. Assay application indicated more frequent detection of 16SrXII-P in samples from Germany and of 16SrXII-A in those from neighboring countries. The assays provide a rapid and accessible tool for subgroup differentiation and may facilitate large-scale epidemiological surveillance and future studies of host tolerance.

## 1. Introduction

Phytoplasmas are small, pleomorphic bacteria of the class Mollicutes associated with diseases in hundreds of plant species worldwide. Affected plant species include many important crops and can lead to growth abnormalities and severe yield losses. Therefore, phytoplasmas represent economically important pathogens in agriculture [1,2]. Phytoplasmas colonize the phloem of their host plants and are transmitted by phloem-sap feeding insects, such as plant- and leafhoppers [3]. Stolbur phytoplasmas represent a heterogeneous 16S rRNA group (16SrXII) [4] within the provisional genus ‘*Candidatus* Phytoplasma’ [5]. The bacteria of the subgroup 16SrXII-A of the taxon ‘*Ca*. Phytoplasma solani’ [6] comprise well-known plant disease agents in Europe, affecting many important crops, including grapevine (*Vitis vinifera*) [6], potato (*Solanum tuberosum*) [7,8,9,10], tomato (*Solanum lycopersicum*) [11,12], carrot (*Daucus carota* subsp. *sativus*) [13], and maize (*Zea mays*) [14,15]. Since the mid-1990s, sugar beet production in Europe has been increasingly affected by two vector-borne bacterial diseases, in which ‘*Ca*. P. solani’ plays distinct roles. Subgroup 16SrXII-A acts as the causal agent of rubbery taproot disease (RTD), predominantly reported in Central Europe [16,17]. In contrast, in the syndrome “basses richesses” (SBR) [18], a disease mainly occurring in Western Europe, particularly in France and Germany, and caused by the γ-proteobacterium ‘*Candidatus* Arsenophonus phytopathogenicus’, it has only been detected as an accompanying pathogen [19,20]. RTD- and SBR- affected beets show similar symptoms, for example, root deformations, chloroses, as well as economically significant reductions in absolute sugar content and biomass losses [16,18,21]. In the context of SBR, another ‘*Ca*. P. solani’-related subgroup, 16SrXII-P, has been identified in SBR-affected sugar beet in Germany. This ribosomal RNA subgroup was first described in sugar beet production areas of the Elbe Valley in eastern Germany, where it was associated with severe yield losses in affected fields [22]. Differences between the phytoplasma subgroups 16SrXII-A and 16SrXII-P, as revealed by genome-wide comparisons, were primarily linked to features involved in pathogen–host interactions, particularly virulence-associated factors [23,24].

The main vector species associated with transmission of ‘*Ca*. P. solani’ subgroup 16SrXII-A in the Central European RTD pathosystem is *Reptalus salicinus* (Tishechkin & Emeljanov 2024), which was formerly known as *R. quinquecostatus* (Dufour, 1833) and confused with *R. artemisiae* (Becker, 1865) [25,26,27,28,29,30]. In the SBR pathosystem, *Pentastiridius leporinus* (Linnaeus, 1761) has been demonstrated as a vector of ‘*Ca*. A. phytopathogenicus’ and ‘*Ca*. P. solani’ subgroup 16SrXII-A to sugar beet in France [6,19,20,31,32]. The subgroup 16SrXII-P has also been detected in *P. leporinus*, while both 16SrXII-A and 16SrXII-P have been reported from SBR-affected sugar beet in Germany [10,22,33,34,35,36]. However, experimental transmission of 16SrXII-P by *P. leporinus* has not yet been demonstrated, and its vector competence remains to be confirmed. In Germany, the nationwide spread of SBR has been accompanied by an expansion of *P. leporinus* populations, with an estimated 119,000 hectares of sugar beet affected, corresponding to approximately one third of the national cultivation area [37]. Over the same period, the total sugar beet area declined from about 380,000 ha in the 2024/25 campaign to around 350,000 ha in 2025/26, representing a reduction of nearly 10%, although this decline cannot be attributed solely to the disease situation [38,39]. These developments underline the increasing agronomic and economic relevance of the disease, particularly in view of its ongoing spread towards previously less affected sugar beet growing regions [37]. As Germany is one of the leading sugar beet producing countries in the European Union, ranking second after France and before Poland [40], further expansion of *P. leporinus*, therefore, poses a substantial threat to German sugar beet production. Given the importance of Germany within the European sugar beet sector, the ongoing spread of the disease complex is also of wider relevance for neighboring production systems. Beyond Germany, 16SrXII-P has also been detected in SBR/RTD-associated samples from other European countries, including Switzerland, Poland, and Austria [17,28,41,42]. Beyond sugar beet, potato has been demonstrated as an additional host of *P. leporinus* [43,44,45,46], while carrot has more recently been suggested as an additional host [47]. Associations with perennial crops such as asparagus (*Asparagus officinalis*) and rhubarb (*Rheum rhabarbarum*) have also been reported [48]. Furthermore, both 16SrXII-A and 16SrXII-P phytoplasmas have been detected in *P. leporinus* collected from RTD-affected sugar beet plots in Austria. Notably, *R. salicinus* was shown to harbor and experimentally transmit both subgroups [28]. Given such overlaps between the SBR and RTD pathosystems and the involvement of different crops and vector systems, disease management is increasingly complicated, underscoring the need for robust diagnostic approaches to differentiate the associated pathogens.

Since the beginning of the SBR outbreaks in France and Germany, the subgroup identity of ‘*Ca*. P. solani’ infections remained unclear for a considerable period, as reports generally referred only to stolbur phytoplasma without further subgroup differentiation, [19,20,43,44]. Hitherto, subgroup characterization in sugar beet and other crops has been restricted to a few selected regions [10,22,36,48]. These findings were further expanded by a recent diversity analysis, which provided new insights into the distribution of stolbur phytoplasma subgroups in German sugar beet production systems and indicated a sporadic occurrence of 16SrXII-A, alongside a higher representation of 16SrXII-P in the investigated samples [35]. However, comparative subgroup analyses based on individual samples across different German production regions are still lacking. Moreover, the most commonly applied molecular approaches for subgroup characterization rely on labor-intensive methods, such as PCR coupled with restriction fragment length polymorphism (RFLP) analysis or Sanger sequencing [17,28,47]. These approaches may reach their limits in the presence of mixed infections, which are particularly relevant for ‘*Ca*. P. solani’, as co-infections with closely related phytoplasmas, such as ‘*Ca*. P. convolvuli’, have been reported [28,49]. However, reliable differentiation of the 16SrXII-A and 16SrXII-P subgroups may support future studies addressing their host associations and epidemiology [27] and could potentially provide additional information relevant to ongoing and future studies on varietal tolerance [50,51].

In this work, we developed a discriminative PCR-based approach targeting the single-copy *secY* gene, encoding the SecY subunit of the phytoplasma *Sec*-dependent secretion system [52], for reliable differentiation of the stolbur phytoplasma subgroups 16SrXII-A and 16SrXII-P. The assays were applied to sugar beet samples from different production regions in Germany and selected neighboring countries. In addition, planthopper samples from corresponding German sugar beet growing regions, as well as red beets and planthoppers from a plot in southern Germany, were analyzed using the newly developed assays to evaluate their applicability across different SBR/RTD-relevant sample types.

## 2. Results

### 2.1. Assay Evaluation and Detection Limit Estimation

**Figure 1:**
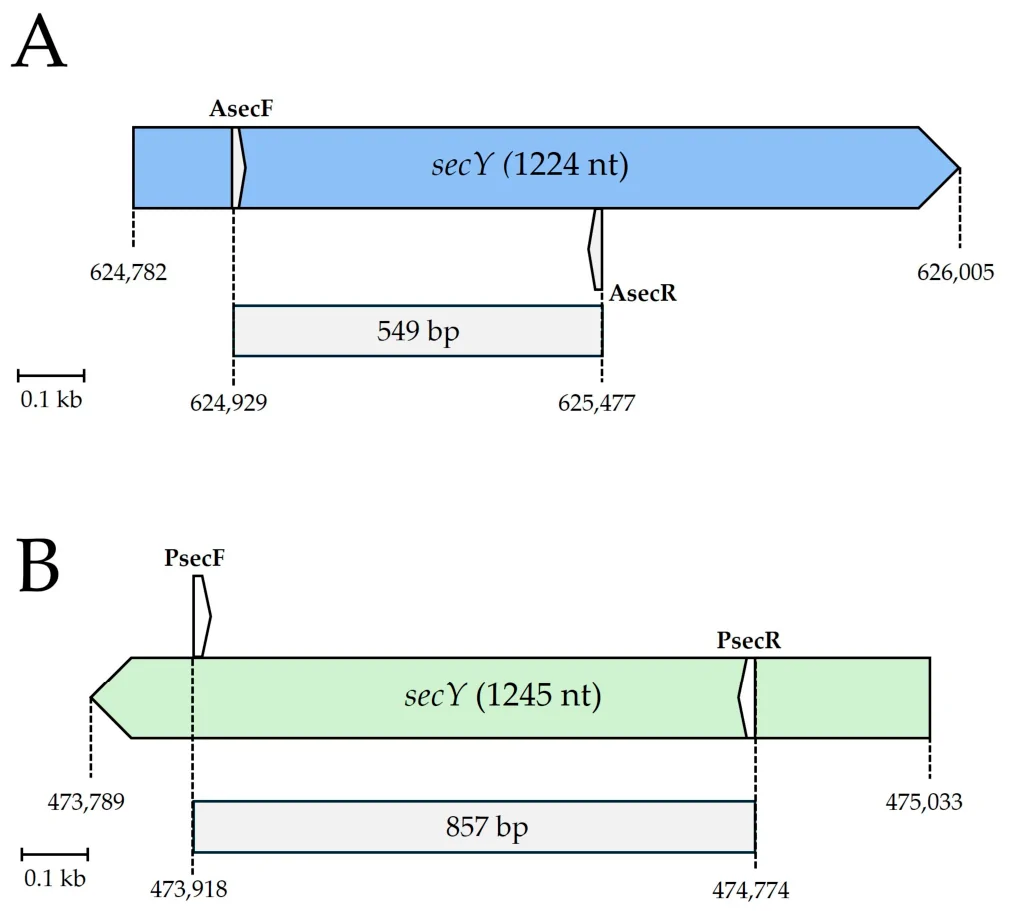
Schematic overview of the in silico predicted *secY* amplicons (grey) used for the differentiation of subgroup 16SrXII-A (blue) with AsecF/AsecR (**A**) and subgroup 16SrXII-P (green) with PsecF/PsecR (**B**), illustrated using the genome sequences of ‘*Ca*. P. solani’ strain c1 (CP103788) and the ‘*Ca*. P. solani’-related strain GOE (CP155828) as representative genomes of the 16SrXII-A and 16SrXII-P subgroups, respectively. Numbers connected by dashed lines indicate the corresponding chromosomal positions. Arrowheads indicate gene length and primer orientation.

In silico analyses of the 16SrXII-A (AsecF/AsecR) and 16SrXII-P (PsecF/PsecR) subgroup-discriminating primers, predicted *secY* amplicons of 549 bp for subgroup 16SrXII-A and 857 bp for subgroup 16SrXII-P, respectively (Figure 1). Predictions with Primer-Basic Local Alignment Searching Tool (BLAST) [53,54] supported the intended targeting of the SecY regions of 16SrXII-A and 16SrXII-P and did not indicate potential off-target amplification in the available nucleotide sequence data for *Beta vulgaris* subsp. *vulgaris*, the class *Insecta*, or other phytoplasma sequences apart from those of subgroups 16SrXII-A and 16SrXII-P. Furthermore, BLAST checks of the AsecF/AsecR primer pair confirmed for both primers a 100% match with all available complete reference genome sequences of the ‘*Ca*. P. solani’ subgroup 16SrXII-A with the strains c1, c4, c5, o3 [55], POT [24], and 135-24 [56]. The same BLAST results were obtained for PsecF/PsecR for ‘*Ca*. P. solani’-related 16SrXII-P subgroup, when compared to the strains GOE [33] and PENLEP [34]. Overall, the in silico analyses supported the intended target specificity and differentiation of the two subgroups.

**Figure 2:**
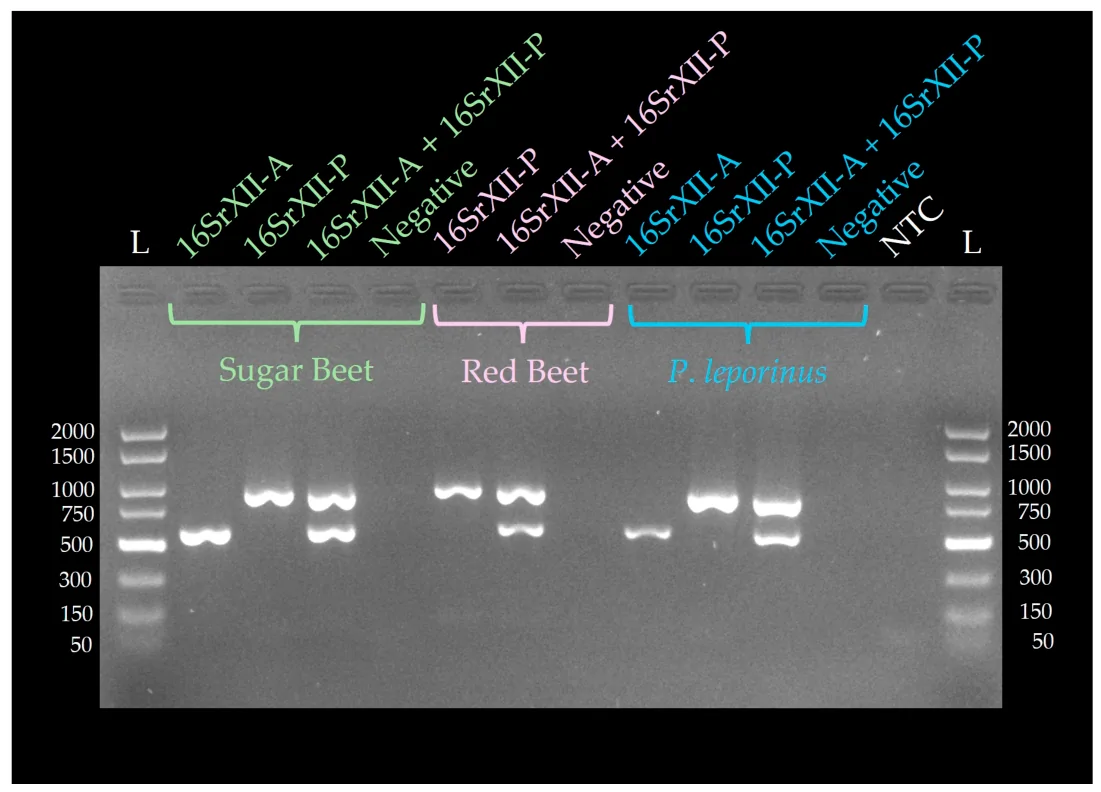
Agarose gel electrophoresis of representative sugar beet (pale green), red beet (pale purple), and *P. leporinus* (pale blue) samples. Bands represent pooled amplicons from individual AsecF/AsecR (549 bp) and PsecF/PsecR (857 bp) PCR reactions. Negative lanes correspond to pathogen-negative sugar beet, red beet, or *P. leporinus* DNA templates. NTC, non-template control (nuclease-free water); L, DNA ladder used for fragment size estimation ranging from 2 kb to 50 bp.

Based on the initial evaluation and subsequent optimization of the PCR conditions, final annealing temperatures of 64 °C for AsecF/AsecR and 60 °C for PsecF/PsecR were established and used for all subsequent analyses. Among the 40 sugar beet samples selected for the initial assay evaluation, 39 (97.50%) tested positive with both the universal P1/P7 [57,58] assay and the *secY*-based differentiation assays using AsecF/AsecR and PsecF/PsecR (Figure S1). P1/P7 amplification within the 40 samples from the RTD-affected Austrian regions yielded 38 (95.00%) positives, while the *secY*-based system provided 39 (97.50%) positives (Figure S2). Concordance between the P1/P7 and *secY*-based assays was 100% in Eppingen from Germany and 95% and 100% in the Austrian fields at Jedenspeigen and Ginzersdorf, respectively. Subgroup differentiation with the sample set from the German SBR-affected regions comprised only single infections with 16SrXII-P, whereas no 16SrXII-A infections or mixed infections were detected. The Austrian sample set comprised 38 (95.00%) single 16SrXII-A infections, no single 16SrXII-P infections, and one mixed infection. Besides sugar beet, the applicability of the differentiation assays was also checked initially on other sample types using selected DNA samples from red beet and *P. leporinus* (Figure 2). Furthermore, no off-target amplification was observed in negative samples containing host-derived DNA from any of the three sample types. Additionally, subgroup affiliation of the generated amplicons from all three sample types was confirmed by Sanger sequencing for selected sequences with both assays (see Section 2.6).

Taken together, the obtained results with AsecF/R and PsecF/R showed broad correspondence to those obtained with the established universal P1/P7 phytoplasma detection system. Furthermore, all amplification products matched the expected in silico predicted sizes of the subgroup-specific *secY* amplicons and clear separation of mixed infections was also obtained for differentiation when used as pooled input for gel electrophoresis. The evaluated sample sets comprised all relevant infection patterns, including single infections with 16SrXII-A, single infections with 16SrXII-P, and mixed infections, enabling the initial evaluation of the subgroup differentiation assays.

**Figure 3:**
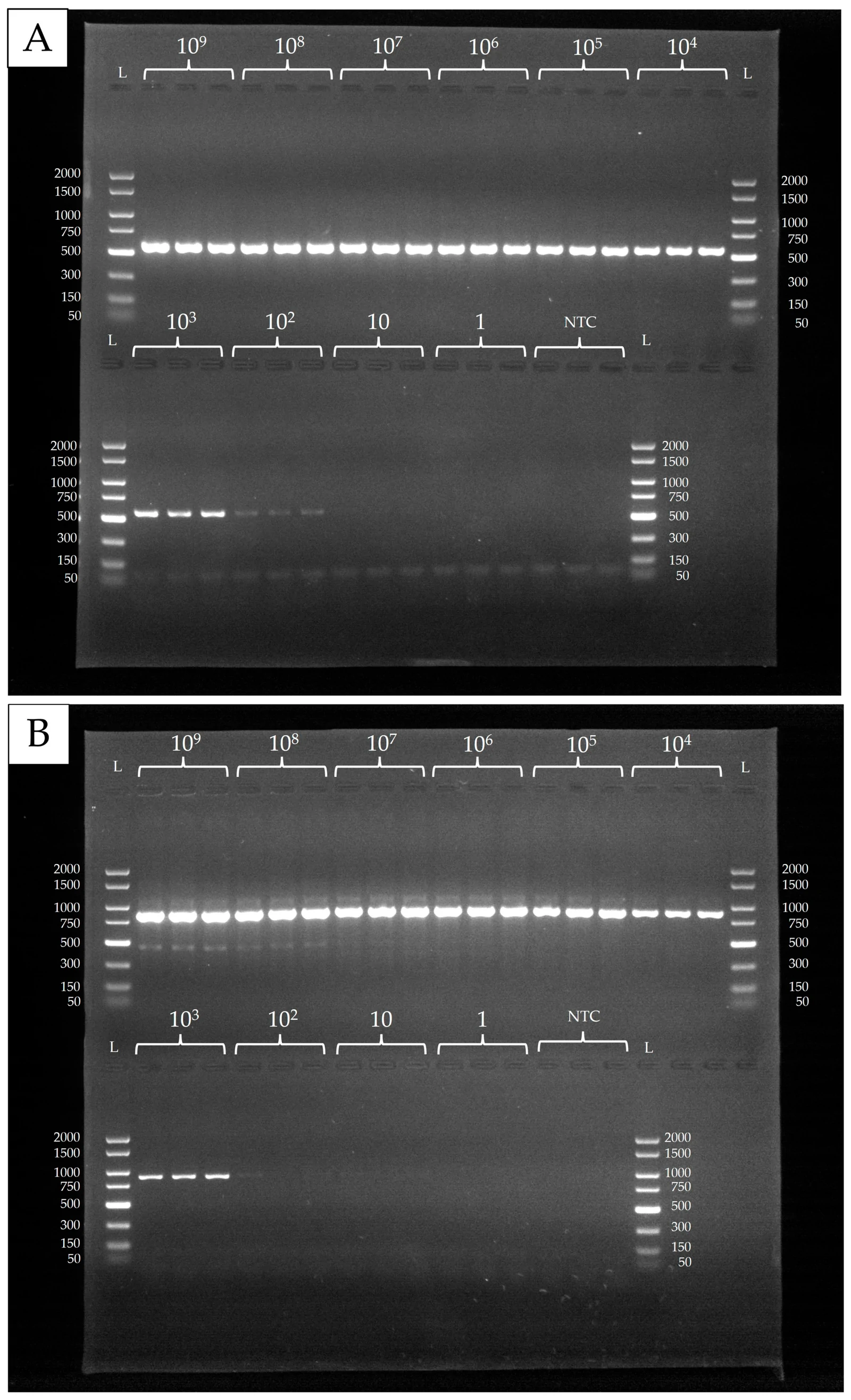
Agarose gel of *secY*-based conventional PCR assays using synthetic gBlock fragments for detection limit estimation of ‘*Ca*. P. solani’ (16SrXII-A) with AsecF/AsecR (**A**) and the related 16SrXII-P subgroup with PsecF/PsecR (**B**). Numbers from 10^9^ to 1 indicate tenfold serial dilutions of synthetic gBlock copy numbers used as input DNA, each tested in triplicate. NTC denotes the non-template control, and L represents the DNA ladder, with fragment sizes indicated next to the bands ranging from 2 kb to 50 bp.

After verification of target-specific amplification and reliable differentiation of both subgroups across different host systems, the detection limits of the assays were estimated. For this purpose, synthetic double-stranded gene fragments (gBlocks) with defined target copy numbers were amplified in triplicate for each assay (Figure 3). The 16SrXII-A-targeting AsecF/AsecR exhibited a lower limit of detection compared to PsecF/PsecR, where the 549 bp-long amplicon was detected in triplicates down to 100 copies (Figure 3A). The detection limit of the PsecF/PsecR assay, which generates a longer 857 bp amplicon, was determined to be 1000 target copies, with consistent amplification observed in all three replicates (Figure 3B).

### 2.2. Detection of 16SrXII-A and 16SrXII-P Phytoplasmas in Sugar Beet and Red Beet

For assessment of the assay performance across phytoplasma populations with differing subgroup compositions [35], 900 sugar beet samples collected during the 2024 harvest campaign were analyzed with our new *secY*-based assay and also compared against the results obtained with the universal P1/P7 assay (Table 1). Detection of 443 (61.53%) phytoplasma-positives was achieved using P1/P7 within the 720 samples from German production areas, while the differentiation assays based on *secY* produced 470 (65.27%). For the 180 samples originating from neighboring countries a total of 43 (23.89%) samples were tested positive with P1/P7, while the *secY* assay yielded 52 (28.89%). Differentiation with the *secY* assays revealed 464 (64.44%) samples from Germany that were tested exclusively positive for the 16SrXII-P subgroup and six samples (0.83%) with both 16SrXII-A and 16SrXII-P were identified, whereas single infections with 16SrXII-A were not detected. In contrast, analysis of the 180 samples from neighboring countries showed 44 (24.44%) positives for subgroup 16SrXII-A-only infections and seven (3.89%) single infections for 16SrXII-P, while only one mixed infection (0.56%) with both subgroups was identified. Concordant results were obtained for 680 (94.44%) of 720 analyzed German sugar beet samples with both assays, while a concordance of 95.00% was observed for 171 of 180 sugar beet samples from the analyzed neighboring countries (Table S1). Within the 50 red beet samples from Stuttgart-Ditzingen in Baden-Wuerttemberg, the universal phytoplasma assay detected 47 (94%) positives using the P1/P7 primers and nested primers R16F2n/R16R2 [58,59] targeting the 16S rRNA gene (Figure S3). Subgroup differentiation with AsecF/AsecR and PsecF/PsecR showed that the majority of the red beets analyzed were positive for 16SrXII-P-only with 40 (80%), and four (8%) showed mixed infections with both subgroups. Similar to the sugar beet samples, concordant results were obtained for 45 (90.00%) of the 50 analyzed red beet samples when comparing both approaches. Taken together, the results obtained with the SecY-based differentiation assays were largely consistent with those obtained using the universal phytoplasma assay targeting the ribosomal operon in both sugar beet and red beet samples. Furthermore, the analyzed dataset revealed contrasting subgroup detection patterns between the German and neighboring-country plots. In the German sugar beet and red beet plots included in this study, 16SrXII-P accounted for the majority of detections, whereas 16SrXII-A was detected only sporadically and exclusively in mixed infections. In contrast, 16SrXII-A showed higher proportions than 16SrXII-P in most of the analyzed plots from neighboring countries.

### 2.3. Regional Patterns of the Subgroups 16SrXII-A and -P in Sugar Beet and Red Beet

**Figure 4:**
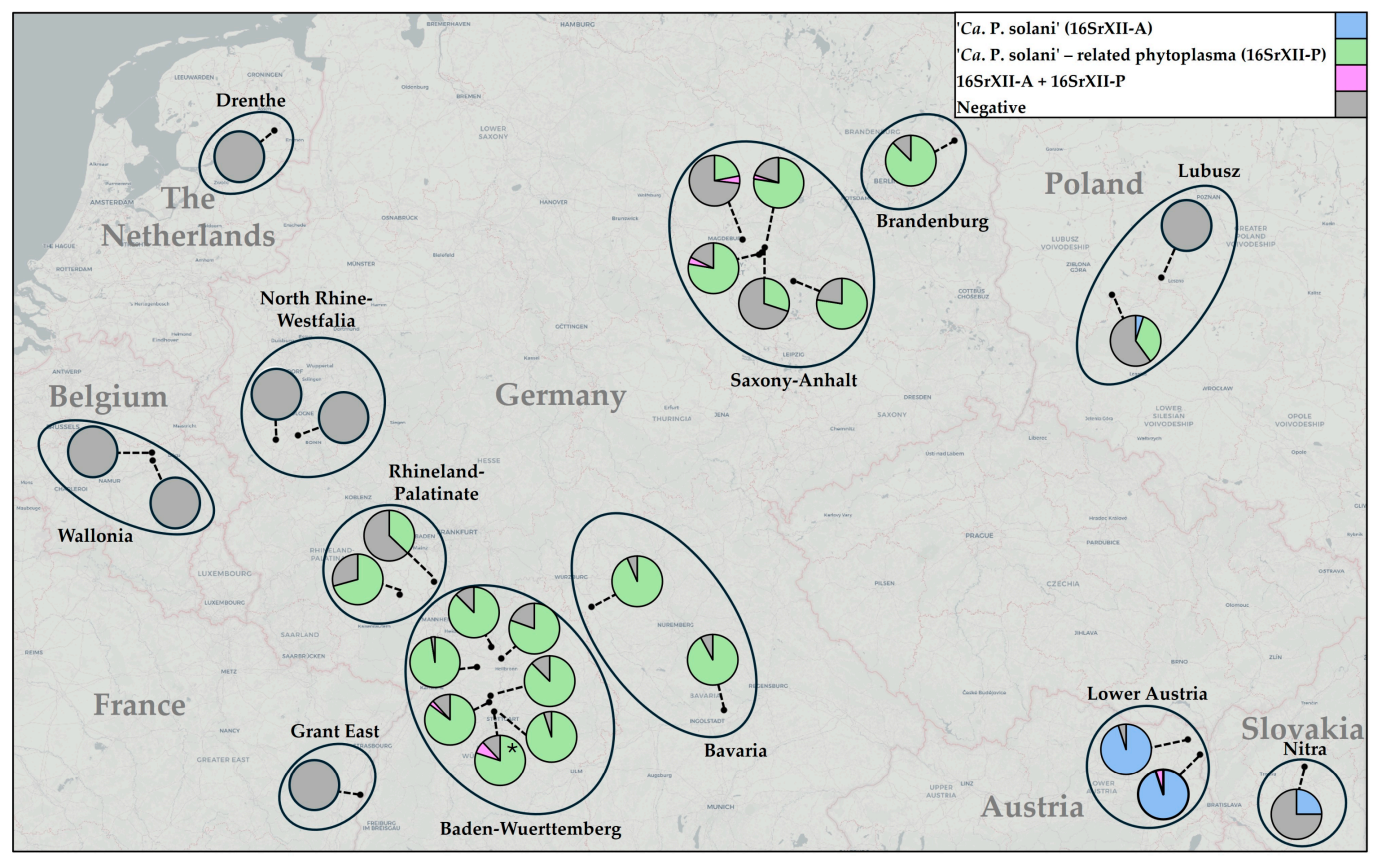
Geographic distribution and relative proportions of ‘*Ca*. P. solani’ subgroup 16SrXII-A (blue), the related subgroup 16SrXII-P (pale green), mixed infections with both subgroups (pink), and negative tested samples (grey) in the analyzed sugar beet templates across Germany and neighboring countries from 2024 indicated as pie charts. Individual sampling sites are shown as black dots, whereas black circles summarize plots at the federal state or regional level. Results obtained from red beet in 2025 are highlighted with an asterisk. The map was generated using QGIS v4.0 (QGIS Development Team) with basemap tiles from CARTO (CartoDB Positron), based on data from OpenStreetMap contributors (https://www.openstreetmap.org/, accessed on 10 March 2026) and CARTO (https://carto.com/, accessed on 10 March 2026). The data shown correspond to those presented in Table 1 and are provided here for spatial visualization.

In the analyzed German plots, 16SrXII-A was detected exclusively in mixed infections with 16SrXII-P and was restricted to three plots in Saxony-Anhalt (Gehrden, Hobeck, and Gommern-Nedlitz) and one plot in Baden-Wuerttemberg (Pulverdingen), where one to two mixed-positive samples represented approximately 2 to 5% of the analyzed plants within individual fields (Figure 4). For the 16SrXII-P subgroup, sugar beet samples from southern Germany in the federal states Baden-Wuerttemberg and Bavaria showed overall the highest numbers of positives with 17 to 39 positives ranging from approximately 45% up to more than 97%. The single field tested from Brandenburg followed with 35 positives representing 87.50%, while plots from Saxony-Anhalt showed higher variation of positives for 16SrXII-P, with 9 to 35 (21–77%), followed by the two fields in Rhineland-Palatinate, showing 13 to 29 positives (37–70%). In contrast, in both plots from North Rhine-Westphalia, no positives were identified at all.

Positives identified in the selected plots from neighboring countries originated from Poland, Austria, and Slovakia. Samples positive exclusively for 16SrXII-A were identified in Austrian fields, exhibiting the highest proportion with 19 samples (95%) for both plots, whereas lower proportions were observed in the Slovakian plots from Malé Zálužie with five positives (25%). The Polish plot from Siedlisko-Dębianka also showed one (5%) 16SrXII-A single infection, while seven (35%) single infections with 16SrXII-P were identified. Mixed infections with both subgroups were only detected in Austria, represented by one sample (5%) from Jedenspeigen. Samples from the Netherlands, Belgium, France, and the plot from Poland in Piotrowice were tested negative.

In summary, the obtained results demonstrate the reliable differentiation and detection of ‘*Ca*. P. solani’ subgroup 16SrXII-A and the related 16SrXII-P subgroup across different sugar beet production areas harboring diverse pathogen populations. The developed assays yielded results consistent with those obtained using the established universal phytoplasma assay based on the ribosomal operon [57,58]. Although not intended as a comprehensive epidemiological assessment, these subgroup detection patterns were broadly consistent with previous reports from Germany and selected neighboring countries [16,17,20,28,35,36].

### 2.4. Screening Results from Planthopper Samples from German Sugar Beet and Red Beet Plots

Of the 296 planthoppers collected from German sugar beet plots, 284 (95.95%) were identified as *P. leporinus* using species-specific primers targeting the cytochrome oxidase I (COI) gene [60]. All 24 planthoppers collected from the red beet plot were identified as *P. leporinus*. Screening with the universal phytoplasma primer pair P1/P7 identified phytoplasma-positives in 167 (56.42%) of the 296 planthopper individuals collected from sugar beet fields in Germany (Table 2), whereas in the red beet-associated planthopper population, 20 (83.33%) were tested positive with the P1/P7 combined with R16F2n/R16R2. Application of the *secY* assays for subgroup differentiation in planthoppers from sugar beet yielded a total of 161 (54.39%) phytoplasma positives with one (0.34%) single infection with 16SrXII-A, 154 (52.03%) positives associated with 16SrXII-P single infections, and six (2.03%) that showed mixed infections with both subgroups. Subgroup differentiation within the planthoppers from red beet revealed, in contrast to the red beet samples, exclusively the presence of the 16SrXII-P subgroup, with 14 positive individuals (58.33%). Overall, the P1/P7 and subgroup-specific AsecF/R and PsecF/R assays showed concordant results for 268 (90.54%) of the analyzed sugar beet samples (Table S2). For the planthoppers analyzed from red beet fields, concordant results were obtained for 18 (75.00%) of 24 samples. Further, 16SrXII-P represented the most frequently detected subgroup among the analyzed phytoplasma-positive planthopper samples, consistent with the detection pattern observed in the analyzed sugar beet samples.

### 2.5. Regional Distribution of 16SrXII-A and -P in Planthopper Samples

**Figure 5:**
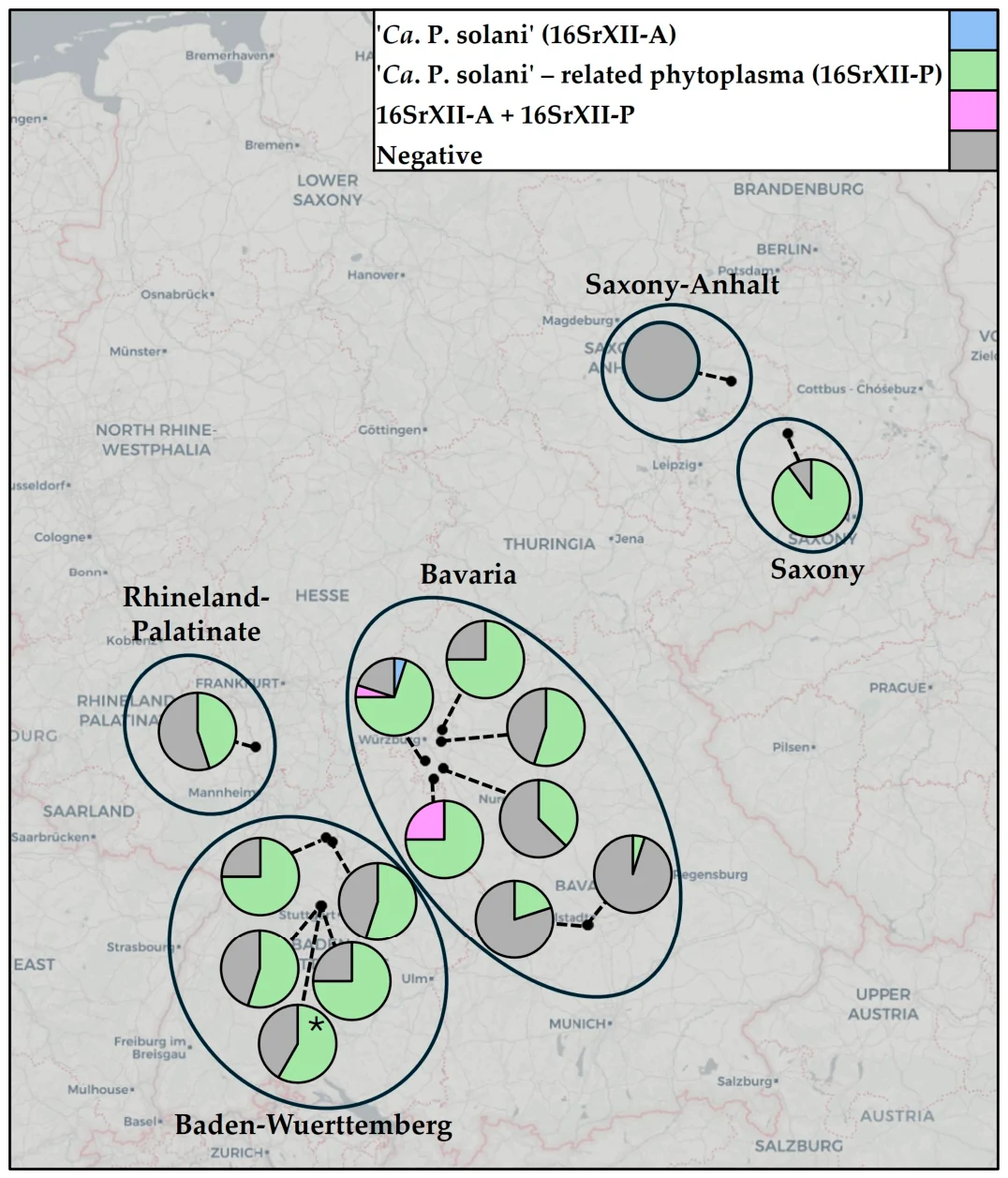
Geographic distribution and relative proportions of ‘*Ca*. P. solani’ subgroup 16SrXII-A (pale blue), ‘*Ca*. P. solani’ related subgroup 16SrXII-P (pale green), mixed infections (pink), and negative tested samples (grey) of the tested planthoppers caught in sugar beet in 2025 across Germany indicated as pie charts. Individual sampling sites are shown as black dots, whereas black circles summarize plots at the federal state level. Results obtained from planthoppers caught in red beet in 2025 are highlighted with an asterisk. The map was generated using QGIS v4.0 (QGIS Development Team) with basemap tiles from CARTO (CartoDB Positron), based on data from OpenStreetMap contributors (https://www.openstreetmap.org, accessed on 10 March 2026) and CARTO (https://carto.com, accessed on 10 March 2026). The data shown correspond to those presented in Table 2 and are provided here for spatial visualization.

The 16SrXII-A subgroup was detected exclusively in planthopper samples from northern Bavaria (Figure 5). Notably, while no single infections with 16SrXII-A were identified in the analyzed sugar beet samples from Germany, one (5%) phytoplasma-positive planthopper identified as *P. leporinus* individual from Giebelstadt carried a single infection with 16SrXII-A (GenBank accession no. PZ581741), whereas another one (5%) harbored a mixed infection with 16SrXII-P. Further mixed infections were restricted to northern Bavaria, with the highest proportion observed in Gelchsheim, where five (25%) of the planthoppers analyzed carried both subgroups. Overall, the highest proportions of 16SrXII-P single infections were observed in samples from Baden-Wuerttemberg, northern Bavaria (Franconia), and eastern Germany (Saxony), ranging from 18 to 20 (50–95%). Planthoppers tested from Kemberg (Saxony-Anhalt) showed no detections in the analyzed sample set. Lower numbers of positives were observed in southern Bavaria and Rhineland-Palatinate, with one to 18 positives leading to proportions starting from 5% up to 45%. Taken together, regional comparison of the subgroup differentiation results obtained from planthopper samples, further supported the applicability of the assay across different sample types and regions. The observed subgroup detection pattern was largely consistent with that observed in the analyzed sugar beet and red beet samples from Germany. Among the phytoplasma-positive planthoppers, 16SrXII-P accounted for the majority of detections, whereas 16SrXII-A was detected only sporadically. Moreover, except for a single sample from Giebelstadt (Bavaria), 16SrXII-A was exclusively detected in mixed infections with 16SrXII-P.

### 2.6. Phylogenetic Clustering with Reference Data Confirms Target-Specificity of the Assays

**Figure 6:**
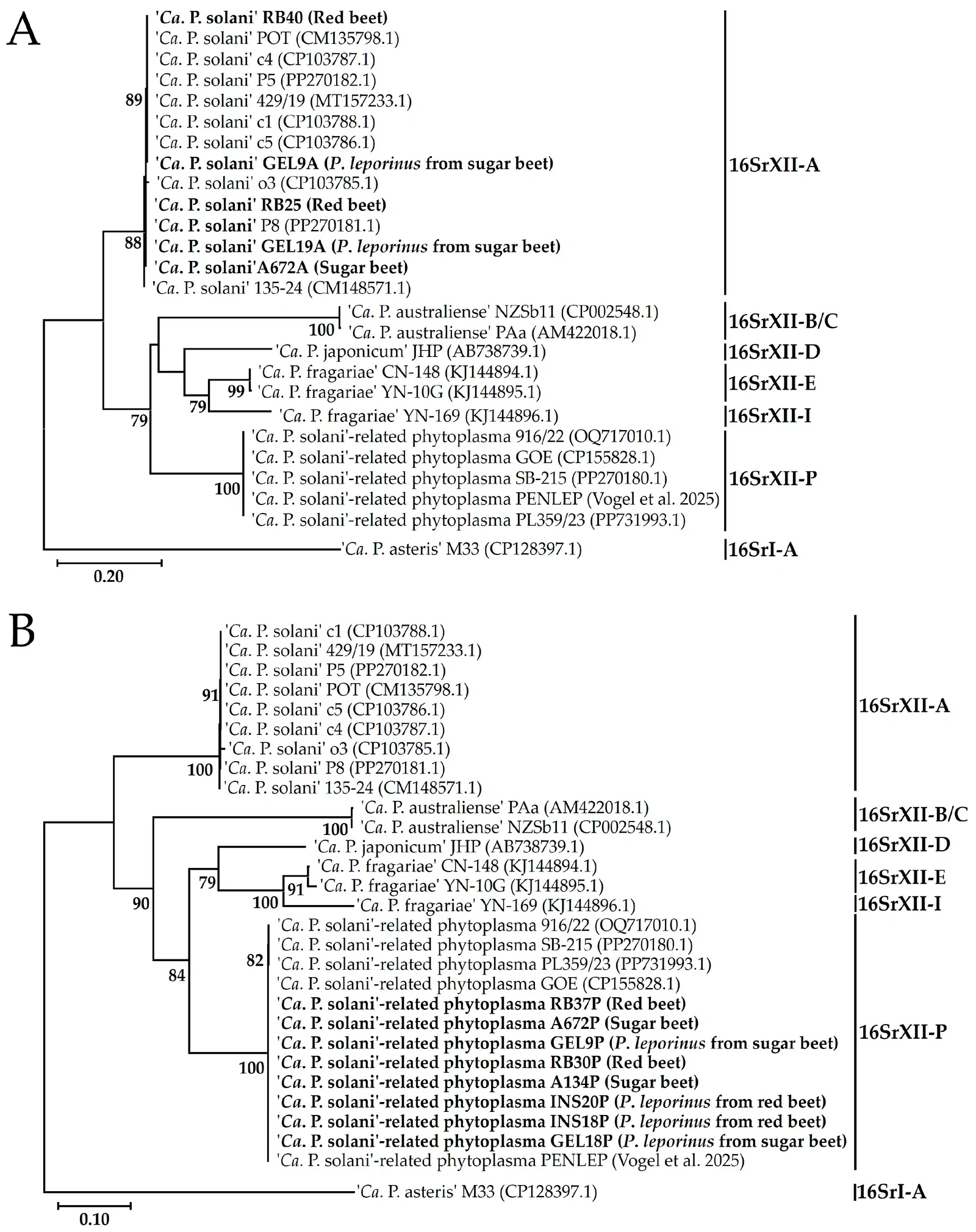
Maximum-likelihood tree of the partial gene *secY* obtained with the AsecF/AsecR (**A**) and PsecF/PsecR (**B**) assays from sugar beet, red beet, and *P. leporinus*, together with selected related stolbur/phytoplasma subgroups. Tree construction was based on multiple sequence alignments comprising 503 and 799 nucleotide positions for the AsecF/AsecR and PsecF/PsecR assays, respectively, and was inferred using 1000 bootstrap replicates. Bootstrap support values ≥ 70% are shown at the corresponding nodes. Branch lengths are drawn to scale and represent the number of substitutions per site. Sequences obtained in this study are highlighted in bold. Reference sequences are indicated by their GenBank accession numbers, while the source publication for PENLEP is indicated in the tree and cited as reference [34].

To further verify the subgroup assignments obtained by the differentiation assays, representative *secY* amplicons of both the 16SrXII-A and 16SrXII-P subgroup were selected from sugar beet, red beet, and *P. leporinus* samples and subjected to Sanger sequencing. Sequence assembly successfully generated partial *secY* consensus sequences for fourteen representative amplicons, with three from sugar beet, four from red beet and seven from *P. leporinus* (Figure 6). BLAST analysis confirmed the subgroup assignments obtained by the differentiation assays, with the 16SrXII-A-derived sequences showing highest similarity to published representatives of the 16SrXII-A subgroup, whereas the 16SrXII-P-derived sequences matched corresponding 16SrXII-P reference sequences. Likewise, maximum-likelihood analysis using publicly available *secY* reference sequences consistently assigned the obtained sequences to their respective subgroup clades. Sequences affiliated with 16SrXII-A clustered together with the reference genomes of ‘*Ca*. P. solani’ strains c1, c4, and c5 from bindweed, o3 from stinging nettle [55], as well as with the reference strains POT [24] and 135-24 [56] originating from *H. obsoletus* caught in potato and *R. salicinus*, respectively. 16SrXII-P sequences grouped with the reference genomes GOE [33] and PENLEP [34] obtained from *P. leporinus* (Figure 6). Selected references from previous studies utilizing the *secY* gene from sugar beet and potato supported the clustering and subgroup affiliation. Representative *secY* sequences generated in this study were deposited in GenBank under accession numbers PZ581737, PZ581744, and PZ581745 (sugar beet), PZ581736, PZ581740, and PZ581743 (red beet), PZ581738, PZ581739, PZ581741, PZ581746, and PZ581747 (*P. leporinus* collected from sugar beet), and PZ581742 (*P. leporinus* collected from red beet).

## 3. Discussion

In this work, we introduced a novel *secY*-targeting endpoint PCR approach suitable for the detection and differentiation of ‘*Ca*. P. solani’ of the 16SrXII-A subgroup and ‘*Ca*. P. solani’-related phytoplasmas of the 16SrXII-P subgroup in sugar beet, red beet, and planthopper samples. Detection rates obtained with the novel subgroup-specific assays were largely concordant with those obtained using the established universal P1/P7 primer pair targeting the 16S rRNA-ITS-23S rRNA region. The latter has been widely applied for phytoplasma detection in several SBR- and RTD-related studies [10,16,17,22,36]. However, in some regions, detection patterns differ between both detection systems, which may be attributable to several factors. First, the ribosomal operon targeted by the P1/P7 primer system is present in two copies in stolbur phytoplasmas, whereas *secY* is a single-copy gene, as shown for the SBR/RTD-relevant complete genome sequences [23,24,33,34,56]. Moreover, sequence variation between ribosomal operon copies has been demonstrated for stolbur phytoplasmas, including sugar beet-associated strains [35]. Furthermore, the universal P1/P7 primer system contains a mismatch to the 16SrXII-P reference genomes GOE [33] and PENLEP [34]. Together with the substantially longer P1/P7 amplicon (approximately 1780–1790 bp), this mismatch may represent a potential factor contributing to amplification. Differences in PCR chemistry, including the use of a Hot Start DNA polymerase for the *secY*-based assays and a Taq/Pfu polymerase mixture for the P1/P7 assay, represent another potential factor contributing to the observed variation between the two detection systems. However, the individual effects of these methodological factors were not experimentally assessed in the present study. Their contribution to the observed differences therefore remains to be determined through more comprehensive diagnostic comparisons. Potential non-specific amplification and cross-reactivity should also be considered when comparing the two approaches, as the universal P1/P7 primer pair may amplify other Mollicutes. For example, amplification of the phytopathogenic *Spiroplasma citri* has been reported in hemp samples [61].

Previously published *secY*-based assays have improved the molecular detection and characterization of stolbur phytoplasmas, particularly for 16SrXII-A [62,63] and the recently described 16SrXII-P subgroup [10]. In contrast, the assays developed in the present study were designed to enable both detection and subgroup differentiation of 16SrXII-A and 16SrXII-P in a single comparative workflow. Additionally, the generated amplicons are suitable for direct Sanger sequencing, allowing independent confirmation of subgroup assignment and subsequent sequence-based analyses. Thus, this approach complements existing *secY*-based methods and provides a rapid and reliable tool for detection and discrimination of both relevant stolbur phytoplasma subgroups occurring in SBR/RTD-affected production areas. The value of targeted differentiation of closely related 16SrXII phytoplasmas has also been demonstrated for ‘*Ca*. P. solani’ and ‘*Ca*. P. convolvuli’, for which *tuf*-based conventional and duplex real-time PCR approaches enabled their differentiation in the shared host field bindweed, including mixed infections [49]. This further highlights the relevance of discriminative approaches for reliably distinguishing closely related 16SrXII phytoplasmas in shared host systems.

Further, our differentiation analysis of both subgroups was consistent with the previously described sporadic occurrence of 16SrXII-A in German sugar beet [35]. In addition, the detection of 16SrXII-A in samples from selected plots in neighboring countries, including Austria and Slovakia, was consistent with previous reports of its occurrence in these regions [17,22,36]. In contrast, the ‘*Ca*. P. solani’-related subgroup 16SrXII-P was detected in all analyzed sugar beet plots in southern and eastern Germany, consistent with its previously reported widespread occurrence in German sugar beet production areas [10,22,35]. In addition, its detection in a plot near Worms (Rhineland-Palatinate) provides further insights into the occurrence of 16SrXII-P in southwestern Germany. Notably, the Polish plots were the only locations among the neighboring countries where 16SrXII-P accounted for the highest proportion within the investigated sample set, as was found in Germany. This is also consistent with previous findings [17,35]. Additionally, our analysis supports the regional patterns identified for both subgroups in a previously published diversity study [35]. However, the previously reported minor occurrence of 16SrXII-P in a Slovakian plot could not be confirmed using our assays, while the sporadic occurrence of this subgroup in the Austrian sample set was confirmed in the present study [28,35]. Moreover, our analysis also provides initial insights into subgroup differentiation in a German red beet growing area where the analyzed plot showed mainly infections with the 16SrXII-P subgroup, as well as mixed infections with the 16SrXII-A subgroup to a minor extent. The observations in red beet fit those from sugar beet affected by SBR and are contrasting to RTD-affected red beet fields in Serbia where ‘*Ca*. P. solani’ 16SrXII-A was reported exclusively [36]. Differentiation analysis of planthopper individuals collected from German sugar beet production areas showed that most phytoplasma-positive planthoppers contained 16SrXII-P as a single infection, whereas all mixed detections of 16SrXII-A and 16SrXII-P were found in the Bavarian fields. In addition, 16SrXII-A was detected as a single infection in one *P. leporinus* sample. These findings extend previous observations from Austria by documenting the occurrence of both phytoplasma subgroups in phytoplasma-positive planthoppers collected in Germany [28]. In contrast, planthoppers collected from red beet were exclusively positive for 16SrXII-P and were all identified as *P. leporinus*. However, the present study was not designed to assess vector competence, since detection of phytoplasma DNA in *P. leporinus* alone does not demonstrate successful transmission. Controlled transmission experiments combined with subgroup differentiation and comprehensive taxonomic assessment of putative vectors are therefore still required to conclusively determine vector competence for 16SrXII-P, as recently addressed for alternative vectors in RTD-related studies [27,28]. This may become particularly important in the context of transmission to alternative host crops and the ongoing adaptation of *P. leporinus* to additional agroecosystems. Especially since recent studies reported crop-associated subgroup patterns, with only 16SrXII-A detected in potato and 16SrXII-P occurring in sugar beet [10]. Although our analysis expands the currently available molecular data on subgroup occurrence in sugar beet production areas, the primary objective of this study was the evaluation of the newly developed discriminative PCR assays rather than a comprehensive epidemiological assessment. Therefore, the observed geographical patterns should be interpreted within the limitations of the investigated sample sets. A broader and more systematic analysis across major SBR-affected sugar beet production areas is still required to robustly assess the regional distribution and epidemiological relevance of the different subgroups. Previous studies mainly focused on the presence of ‘*Ca*. P. solani’ without differentiation between the respective subgroups [44,50,64,65]. In addition, newly affected production areas emerging with the ongoing spread of *P. leporinus* should also be considered [37]. Moreover, the occurrence of distinct subgroup patterns in different host plants and their potential association with different vector systems [10,17,27,44,47,48,50] underline the value of subgroup-specific differentiation that could support future epidemiological monitoring of overlapping phytoplasma populations in both host plants and vectors.

The limited detection of 16SrXII-P in some regions may also highlight the sensitivity constraints of such endpoint PCR-based approaches. As the analytical detection limits were estimated using synthetic templates only, further evaluation using naturally infected plant and insect samples would further improve the assessment of assay sensitivity under biological conditions. Since the assays were primarily developed to independently confirm the regional 16SrXII-A and 16SrXII-P patterns identified in our preceding diversity analysis [35], additional experimental specificity testing against other relevant phytoplasma groups would further support their application beyond this targeted differentiation context.

To address the sensitivity and quantitative limitations of the present endpoint PCR approach, the development of a corresponding Real-Time PCR assay represents an important next step. A TaqMan-based system could provide a basis for simultaneous differentiation and quantification of 16SrXII-A and 16SrXII-P, complementing currently available Real-Time PCR approaches [45,65,66], which allow sensitive detection of both subgroups but cannot distinguish between them. Such an approach would be particularly valuable for detecting low-abundance infections and comparing subgroup-specific pathogen loads across different sample types. Moreover, future studies could evaluate the applicability of the presented differentiation approach in field-based SBR research trials [50,51]. This could be particularly useful in combination with targeted genotyping of subgroup and strain patterns. In addition, together with more comprehensive characterization data, such information may also be relevant for addressing cultivar responses and varietal tolerance.

## 4. Materials and Methods

### 4.1. Samples from Sugar Beet and Red Beet

During the 2024 harvest campaign, a total of 900 taproot tip samples, with at least 20 samples per plot, were collected between July and November from sugar beet plants exhibiting SBR/RTD-like symptoms in Germany and neighboring countries (Table 3). Of these, 720 were collected from eighteen sugar beet plots, comprising six fields in Baden-Wuerttemberg, two in Bavaria, five in Saxony-Anhalt, one in Brandenburg, and two in each of North Rhine-Westphalia and Rhineland-Palatinate. Outside Germany, 180 samples were collected from nine plots, with one plot sampled in France, Slovakia, and the Netherlands and two plots sampled in Belgium, Austria, and Poland. All samples represented a subset of a previously characterized dataset, in which phytoplasma occurrence and diversity profiles were determined [35]. The dataset was selected to include samples from major SBR-affected production regions as well as regions at the margins of the affected areas, allowing assay performance to be evaluated across different sampling regions. Additionally, 40 symptomatic and ten non-symptomatic taproot tip samples with unknown phytoplasma infection status were collected in August 2025 from a red beet field in Ditzingen located in a severely SBR-affected area near Stuttgart in Baden-Wuerttemberg.

### 4.2. Planthopper Samples

For the analysis in 2025, 296 planthopper individuals, with at least fifteen samples per plot, were collected by netting from May to September from fourteen sugar beet plots in SBR-affected German regions (Table 4): four located in Baden-Wuerttemberg, seven in Bavaria, and one each in Rhineland-Palatinate, Saxony, and Saxony-Anhalt. Sampling focused on planthoppers visually considered potential cixiid vector candidates, without prior taxonomic identification. A total of 200 samples originated from a previously described sample collection [43], which was expanded by an additional 96 samples from Biberach, Seligenstadt, Giebelstadt, Ochsenfurt, and Biebelsried to increase the sample size. Moreover, an additional 24 planthoppers were collected by netting from the severely affected red beet field in Stuttgart-Ditzingen described above. The phytoplasma infection status of all samples was unknown prior to the present analysis.

### 4.3. Extraction of Nucleic Acids from Plant and Insect Material

Nucleic acids were extracted from 1 g of sugar beet or red beet taproot tip material and whole planthopper individuals. For sugar beet and planthopper catches from sugar beet plots, the High Pure PCR Template Preparation Kit (Roche Diagnostics GmbH, Mannheim, Germany) was utilized for extraction, while for red beet samples and planthopper catches from red beet plots the GeneMATRIX Plant & Fungi DNA Purification Kit (EUR_X_ Sp. z. o.o., Gdańsk, Poland) was used. Maceration of the plant samples was performed with 4 mL lysis buffer of the respective kit in plastic extraction bags (Bioreba AG, Reinach, Switzerland) with a semi-automated HOMEX 7 tissue homogenizer (Bioreba AG, Reinach, Switzerland). For insect material, a total of 800 µL tissue lysis buffer was added and macerated with five silica beads at 8 m/s for 90 s in a FastPrep-24 5G bead beating grinder (MP Biomedicals Germany GmbH, Eschwege, Germany). After maceration, for both kits, samples were processed according to the protocol steps provided by the vendor. DNA extraction batches were routinely checked by fluorometric quantification of representative DNA extracts using a Qubit fluorometer (Thermo Fisher Scientific, Waltham, MA, USA). All obtained templates were stored at −20 °C for subsequent analysis.

### 4.4. Primer Design and PCR Conditions

Two discriminative primer pairs were designed for the differentiation of the ‘*Ca*. P. solani’ subgroup 16SrXII-A (AsecF/AsecR) and the subgroup 16SrXII-P (PsecF/PsecR) based on the single-copy gene *secY*, coding for the SecY subunit of the preprotein translocon of the Sec-dependent secretion system of phytoplasmas. In silico analyses predicted *secY* amplicons of 549 bp for subgroup 16SrXII-A and 857 bp for subgroup 16SrXII-P, respectively. As a basis for discriminative primer design, full-length *secY* sequences from all available complete genomes of stolbur phytoplasma subgroups 16SrXII-A represented by the strains c1 (CP103788.1), c4 (CP103787.1), c5 (CP103786.1), and o3 (CP103785.1) [55], as well as for the 16SrXII-P subgroup with the strains GOE (CP155828.1) [33] and PENLEP [34] were retrieved from the GenBank database at National Center for Biotechnology Information (NCBI) (https://www.ncbi.nlm.nih.gov/; accessed on 15 July 2025) and compared using a multiple nucleotide sequence alignment generated in Molecular Evolutionary Genetic Analysis (MEGA) v12, applying the MUSCLE v3.8 algorithm [67] to identify suitable sequence regions enabling discrimination between both subgroups. Novel oligonucleotides were designed using the online service of Primer3 Plus [68] and evaluated with the Primer Basic Local Alignment Searching Tool (BLAST) v.2.17.0 from NCBI [53,54] against the nucleotide (nt) database (https://www.ncbi.nlm.nih.gov/tools/primer-blast/; accessed on 10 August 2025) for target specificity. Potential off-target amplification of the phytoplasma hosts was assessed in silico against the nt data of *Beta vulgaris* subsp. *vulgaris* (taxid: 3555), *Insecta* (taxid: 50557), and other phytoplasmas (taxid 33926) via Primer BLAST. To ensure reliable differentiation of both subgroups on a single gel, only primer pairs were selected whose amplicon lengths differed by at least 250 bp. To cover the 16SrXII-A subgroup, the *secY*-targeting forward primer PosecF3 [62] was utilized and modified at the 3’-end to improve target specificity and was renamed to AsecF. A corresponding reverse primer (AsecR) was newly designed using Primer3Plus. For 16SrXII-P, the novel primer pair PsecF/PsecR was designed using Primer3Plus. All candidate oligonucleotides were synthesized by a local vendor (Metabion International AG, Planegg, Germany) and supplied as 100 µM stock solutions, which were aliquoted and diluted to 10 µM working solutions for subsequent PCR reactions.

End-point PCR assays for the two novel primer pairs AsecF/AsecR and PsecF/PsecR (Table 5) were evaluated in 15 µL reaction volumes, each containing 0.2 µM of each primer, 7.5 µL of OneTaq Hot Start 2X Master Mix with Standard Buffer (New England Biolabs GmbH, Frankfurt am Main, Germany), and 0.15 µL of 10X Color Load Buffer (EUR_X_ Sp. z o.o., Gdańsk, Poland). For each reaction, 4 µL of extracted DNA was used as template, corresponding to approximately 10 to 20 ng based on representative fluorometric measurements, with nuclease-free water added to reach the final reaction volume. For evaluation of optimal PCR conditions, annealing temperatures ranging from 55 to 72 °C were tested with cycling conditions consisting of an initial denaturation at 94 °C for 30 s, followed by 35 cycles of denaturation at 94 °C for 30 s, annealing for 30 s and extension at 68 °C for 1 min, with a final extension at 68 °C for 5 min and subsequent cooling at 8 °C. Optimal annealing temperatures used for screening with AsecF/AsecR were determined at 64 °C and 60 °C for PsecF/PsecR. Each PCR run included a negative control in which nuclease-free water was added instead of DNA template. As a positive control for the 16SrXII-A assay, DNA extracted from *Catharanthus roseus* experimentally infected with the 16SrXII-A reference strain STOL [4], provided by Erich Seemüller (Julius Kühn Institute, Federal Research Centre for Cultivated Plants, Institute for Plant Protection in Fruit Crops and Viticulture, Dossenheim, Germany), was used. For the 16SrXII-P assay, DNA extracted from a 16SrXII-P-infected sugar beet taproot obtained in a previous study was used as a positive control [35]. Both assays were performed separately for each sample. Subsequently, 5 µL of each PCR product were combined and the resulting 10 µL were analyzed by agarose gel electrophoresis, allowing simultaneous evaluation of both assays. PCR products were separated on a 1% agarose gel stained with SafeGel red stain 10,000× in double distilled water (Genaxxon Bioscience GmbH, Ulm, Germany) and visualized under UV illumination. Final target-specific amplification was confirmed by Sanger sequencing from both directions for selected PCR products using a commercial sequencing service provider (Macrogen Europe B.V., Amsterdam, The Netherlands). Obtained read data was assembled using Pregap4 v1.6-r using a shotgun assembly approach with default settings, while successful assemblies were inspected, corrected based on quality, and trimmed manually using Gap4 v4.11.2-r [69]. Final contiguous sequences were checked for taxonomic affiliation using BLAST v2.17.0 against the NCBI nucleotide collection (nt) database (last access 24 June 2026) with the megablast algorithm.

To initially evaluate the *secY*-based assays for the detection and differentiation of 16SrXII-A and 16SrXII-P, two representative sets of sugar beet DNA samples were selected from previously characterized SBR/RTD-affected regions showing contrasting occurrence of the two subgroups. These samples originated from a previously analyzed dataset in which no other 16SrXII subgroups had been detected [35]. The first set comprised 40 DNA samples originating from Germany with samples from Eppingen in Baden-Wuerttemberg (Figure S1), where 16SrXII-P represented the predominant phytoplasma subgroup, whereas the second set consisted of 40 DNA samples from Austrian sugar beet fields from Jedenspeigen and Ginzersdorf (Figure S2) affected by RTD, where 16SrXII-A phytoplasmas dominate the epidemic. Furthermore, selected DNA templates from sugar beet, red beet, and *P. leporinus* samples previously tested as phytoplasma-positive or phytoplasma-negative using P1/P7 [57,58] followed by nested R16F2n/R16R2 PCR [58,59] targeting the 16S rRNA-ITS-23S rRNA locus were used to assess potential off-target amplification from the different sample matrices. *P. leporinus* affiliation of the collected planthopper samples was verified with species-specific primers targeting the cytochrome oxidase I gene [60], as described previously [35].

### 4.5. Sample Screening and Comparison of Detection Rates

For the differentiation of 16SrXII-A and 16SrXII-P phytoplasmas, the DNA samples of all samples described were analyzed using the optimal PCR conditions described above. As a further evaluation step, the novel *secY*-based differentiation system was compared to an established assay for universal phytoplasma detection with the primer pair P1/P7 [57,58]. Since direct P1/P7 amplification in red beet and associated planthoppers occasionally resulted in double-band patterns, nested PCR with the primers R16F2n/R16R2 [58,59] was applied to ensure specific detection. P1/P7 direct PCR in sugar beet and associated planthoppers was performed as described previously for sugar beet [35]. For direct PCR with P1/P7 and subsequent nested PCR with R16F2n/R16R2 using red beet and associated planthopper DNA templates, reaction volumes of 16 µL and 20 µL, respectively, were prepared with OptiTaq PCR Master Mix 2X (EURX Sp. z o.o., Gdańsk, Poland) and performed as described previously [35,58]. To assess the consistency of detection results obtained with the P1/P7 assay and the AsecF/R and PsecF/R assays, detection concordance was determined for all analyzed samples (Tables S1 and S2). Concordance was calculated as the proportion of samples yielding concordant positive or negative results relative to the total number of samples analyzed.

### 4.6. Estimation of Detection Limits

To estimate the detection limits of the *secY*-based differentiation assays, in silico amplicon sequences of AsecF/AsecR and PsecF/PsecR were used to design synthetic double-stranded DNA fragments (gBlocks), which were synthesized by Integrated DNA Technologies Inc. (Coralville, IA, USA). Based on the fragment-specific concentration and molecular weight provided by the manufacturer, the required concentrations were calculated according to the manufacturer’s protocol (https://eu.idtdna.com/page/support-and-education/decoded-plus/tips-for-working-with-gblocks-gene-fragments/, accessed on 10 April 2026). For each gBlock, stock solutions corresponding to an input of 1 × 10^10^ copies per reaction were prepared. A serial dilution series ranging from 1 × 10^9^ copies to 1 copy per reaction was subsequently prepared for each gBlock. Each gBlock dilution series was tested independently, with each dilution step analyzed in three technical replicates. The detection limits were estimated as the lowest copy number yielding a detectable amplification product in all three replicates. Obtained amplicons were visualized on a 1% agarose gel as described above.

### 4.7. Comparison of secY Sequences with Reference Data

To confirm targeted specificity of the developed PCR assays, selected amplicons from representative sugar beet, red beet, and *P. leporinus* samples for both subgroups were directed to Sanger sequencing as described above (Section 4.4). Obtained contiguous sequences were compared using BLAST against the NCBI nt database with the megablast v2.16.0. algorithm to confirm target specificity and subgroup affiliation. Representative sequences were selected for phylogenetic clustering with reference *secY* sequences from sugar beet and potato (Table S2) and complete reference genomes representing ‘*Ca*. P. solani’ subgroup 16SrXII-A, including strains c1 (CP103788.1), c4 (CP103787.1), c5 (CP103786.1), o3 (CP103785.1), POT (CM135798.1), and 135-24 (CM148571.1), and the ‘*Ca*. P. solani’-related subgroup 16SrXII-P, including strains GOE (CP155828.1) and PENLEP [34]. Additionally, stolbur phytoplasma reference genomes representing the 16SrXII-B/C subgroup of ‘*Ca*. P. australiense’ with the strains PAa (AM422018.1) and NZSb11 (CP002548.1), and sequences of the subgroups 16SrXII-D of ‘*Ca*. P. japonicum’, as well as of 16SrXII-E and 16SrXII-I of ‘*Ca*. P. fragariae’ (Table S2), were included for robust phylogenetic clustering. Moreover, the *secY* sequence of ‘*Ca*. P. asteris’ strain M33 (CP128397.1) [70] was included as an outgroup reference for phylogenetic analyses. For clustering, nucleotide sequences were aligned using the MUSCLE algorithm implemented in MEGA v12 [67] under default settings. Phylogenetic tree constructions were performed using the maximum-likelihood approach in IQ-TREE v2.4.0 [71], applying the best-fitting substitution model selected according to the Bayesian information criterion (BIC). Node support was evaluated by 1000 bootstrap replicates, with values ≥ 70% considered statistically supported. Phylogenetic trees were visualized and edited in MEGA.

## 5. Conclusions

Here, we provide a novel discriminative endpoint PCR approach based on the SecY gene for differentiation between the ‘*Ca*. P. solani’ subgroup 16SrXII-A and the related 16SrXII-P subgroup in samples from sugar beet, red beet, and associated planthoppers. The evaluation of the developed assay demonstrated its applicability for subgroup differentiation across all analyzed sample types from selected SBR/RTD-affected plots in Germany and neighboring countries. Within the analyzed German sugar beet samples, 16SrXII-P accounted for the majority of detections, whereas 16SrXII-A was detected only sporadically. In contrast, 16SrXII-A showed higher proportions than 16SrXII-P among the analyzed sugar beet samples from selected plots of neighboring countries. Overall, this approach provides a practical basis for further investigation of 16SrXII-A and 16SrXII-P in SBR/RTD-associated plant and insect material and may support future studies on subgroup distribution and disease monitoring, while potentially providing additional information for studies addressing cultivar responses and tolerance.

## Figures and Tables

**Table 1:** Phytoplasma screening in Germany and neighboring countries.

Sample Source	Country	Federal State/Region	Location	Sample No. *n*	Universal Phytoplasma Assay (P1/P7)	Subgroup Differentiation Assays (AsecF/R + PsecF/R)			
					Positives *n* (%)	Positives *n* (%)	16SrXII-A *n* (%)	16SrXII-P *n* (%)	Mixed *n* (%)
Sugar beet	Germany	Baden-Wuerttemberg	Bad Wimpfen Hueffenhardt Eppingen Oberriexingen Ditzingen Pulverdingen /Markgröningen	41 40 40 40 40 34	26 (63.41) 34 (85.00) 39 (97.50) 31 (77.50) 35 (87.50) 25 (73.52)	33 (80.49) 35 (87.50) 39 (97.50) 35 (87.50) 38 (95.00) 30 (88.24)	0 0 0 0 0 0	33 (80.49) 35 (87.50) 39 (97.50) 35 (87.50) 38 (95.00) 29 (85.30)	0 0 0 0 0 1 (2.94)
		Bavaria	Rodheim Rain am Lech/Oberdolling	44 38	43 (97.73) 14 (36.84)	43 (97.73) 17 (44.74)	0 0	43 (97.73) 17 (44.74)	0 0
		Saxony-Anhalt	Gehrden Hobeck Gommern-Nedlitz Vockerode Buhlendorf	45 40 41 40 40	36 (80.00) 31 (77.50) 11 (26.83) 30 (75.00) 12 (30.00)	37 (82.22) 32 (80.00) 11 (26.83) 31 (77.50) 12 (30.00)	0 0 0 0 0	35 (77.78) 31 (77.50) 9 (21.95) 31 (77.50) 12 (30.00)	2 (4.44) 1 (2.50) 2 (4.88) 0 0
		Brandenburg	Neureetz	40	34 (85.00)	35 (87.50)	0	35 (87.50)	0
		Rhineland-Palatinate	Worms-Eich Bischheim	41 35	29 (70.73) 13 (37.14)	29 (70.73) 13 (37.14)	0 0	29 (70.73) 13 (37.14)	0 0
		North Rhine-Westphalia	Bornheim Vettweiß	41 40	0 0	0 0	0 0	0 0	0 0
		Total *n* (%)		720	443 (61.53)	470 (65.27)	0	464 (64.44)	6 (0.83)
Red beet		Baden-Wuerttemberg	Ditzingen	50	47 (94.00) ^1^	44 (88.00)	0	40 (80.00)	4 (8.00)
Sugar beet	France	Great East	Richtolsheim	20	0	0	0	0	0
	Austria	Lower Austria	Ginzersdorf Jedenspeigen	20 20	19 (95.00) 19 (95.00)	19 (95.00) 20 (100.00)	19 (95.00) 19 (95.00)	0 0	0 1 (5.00)
	Slovakia	Nitra	Malé Zálužie	20	1 (5.00)	5 (25.00)	5 (25.00)	0	0
	Poland	Lubusz	Siedlisko-Dębianka Piotrowice	20 20	4 (20.00) 0	8 (40.00) 0	1 (5.00) 0	7 (35.00) 0	0 0
	The Netherlands	Drenthe	Orvelte	20	0	0	0	0	0
	Belgium	Wallonia	Verlaine Donceel	20 20	0 0	0 0	0 0	0 0	0 0
		Total *n* (%)		180	43 (23.89)	52 (28.89)	44 (24.44)	7 (3.89)	1 (0.56)

^1^ Phytoplasma-positive samples refer to the results obtained by subsequent analysis using the universal primer pairs P1/P7 and R16F2n/R16R2. Percentages represent the proportion of the total sample amount.

**Table 2:** Phytoplasma screening results for planthopper samples from Germany.

Sample Source	Federal State	Location	Sample No. *n*	*P. leporinus* *n* (%)	Universal Phytoplasma Assay (P1/P7)	Subgroup Differentiation Assays (AsecF/R + PsecF/R)			
					Positives *n* (%)	Positives *n* (%)	16SrXII-A *n* (%)	16SrXII-P *n* (%)	Mixed *n* (%)
Sugar beet field	Baden-Wuerttemberg	Ditzingen Hirschlanden Treschklingen Biberach	20 20 20 20	20 (100.00) 20 (100.00) 20 (100.00) 19 (95.00)	10 (50.00) 10 (50.00) 15 (75.00) 13 (65.00)	11 (55.00) 15 (75.00) 15 (75.00) 11 (55.00)	0 0 0 0	11 (55.00) 15 (75.00) 15 (75.00) 11 (55.00)	0 0 0 0
	Bavaria	Seligenstadt Giebelstadt Ochsenfurt Biebelsried Gelchsheim Ingolstadt	20 20 16 20 20 20 20	16 (80.00) 19 (95.00) 12 (75.00) 19 (95.00) 20 (100.00) 20 (100.00) 20 (100.00)	17 (85.00) 14 (70.00) 11 (68.75) 15 (75.00) 20 (100.00) 1 (5.00) 4 (20.00)	15 (75.00) 16 (80.00) 6 (37.50) 11 (55.00) 20 (100.00) 1 (5.00) 4 (20.00)	0 1 (5.00) 0 0 0 0 0	15 (75.00) 14 (70.00) 6 (37.50) 11 (55.00) 15 (75.00) 1 (5.00) 4 (20.00)	0 1 (5.00) 0 0 5 (25.00) 0 0
	Saxony	Arzberg	20	20 (100.00)	19 (95.00)	18 (90.00)	0	18 (90.00)	0
	Saxony-Anhalt	Kemberg	20	20 (100.00)	0	0	0	0	0
	Rhineland-Palatinate	Worms-Eich	40	37 (92.50)	18 (45.00)	18 (45.00)	0	18 (45.00)	0
Total *n* (%)			296	284 (95.95)	167 (56.42)	161 (54.40)	1 (0.34)	154 (52.03)	6 (2.03)
Red beet field	Baden-Wuerttemberg	Ditzingen	24	24 (100.00)	20 (83.33) ^1^	14 (58.33)	0	14 (58.33)	

^1^ Phytoplasma-positive samples refer to the results obtained by subsequent analysis using the universal primer pairs P1/P7 and R16F2n/R16R2. Percentages represent the proportion of the total sample amount.

**Table 3:** Sugar beet samples with SBR/RTD symptoms collected in Germany and neighboring countries in 2024 and red beet samples collected in Germany in 2025.

Country	Federal State/Region	Location	Geo Coordinates	Sample No.
Germany	Baden-Wuerttemberg	Bad Wimpfen Hueffenhardt Eppingen Oberriexingen Ditzingen Ditzingen * Pulverdingen/Markgröningen	49°12′33.7″ N 9°09′28.6″ E 49°17′39.2″ N 9°03′09.2″ E 49°08′52.9″ N 8°53′40.7″ E 48°56′38.6″ N 9°02′28.8″ E 48°49′48.1″ N 9°04′53.3″ E 48°50′21.0″ N 9°00′47.7″ E 48°53′53.3″ N 9°01′43.0″ E	41 40 40 40 40 50 34
	Bavaria	Rodheim Oberdolling/Rain am Lech	49°34′49.3″ N 10°09′05.9″ E 48°50′33.7″ N 11°36′38.9″ E	44 38
	Saxony-Anhalt	Gehrden Hobeck Gommern-Nedlitz Vockerode Buhlendorf	52°01′09.0″ N 11°59′30.3″ E 52°04′00.3″ N 12°03′17.7″ E 52°07′14.2″ N 11°48′45.2″ E 51°50′22.7″ N 12°22′24.4″ E 52°02′08.5″ N 12°01′35.8″ E	45 40 41 40 40
	Brandenburg	Neureetz	52°47′01.1″ N 14°08′26.7″ E	40
	Rhineland-Palatinate	Worms-Eich Bischheim	49°45′29.1″ N 8°25′31.2″ E 49°40′07.1″ N 8°02′53.5″ E	41 35
	North Rhine-Westphalia	Bornheim Vettweiß	50°47′08.2″ N 6°55′36.9″ E 50°45′15.3″ N 6°40′48.6″ E	41 40
France	Great East	Richtolsheim	48°13′13.3″ N 7°36′48.3″ E	20
Austria	Lower Austria	Ginzersdorf Jedenspeigen	48°37′39.1″ N 16°42′06.2″ E 48°31′15.8″ N 16°50′19.5″ E	20 20
Slovakia	Nitra	Malé Zálužie	48°25′45.6″ N 17°59′15.5″ E	20
Poland	Lubusz	Siedlisko-Dębianka Piotrowice	51°44′46.2″ N 15°51′58.7″ E 51°51′56.7″ N 16°24′34.2″ E	20 20
The Netherlands	Drenthe	Orvelte	52°51′07.1″ N 6°39′51.4″ E	20
Belgium	Wallonia	Verlaine Donceel	50°36′41.8″ N 5°19′55.6″ E 50°39′48.3″ N 5°19′16.4″ E	20 20

Location highlighted with an asterisk represents the tested red beet plot.

**Table 4:** Overview of sampled areas of sugar beet from Germany for planthopper screening from 2025.

Sample Origin	Federal State	Location	GPS-Coordinates	Sample No.
Sugar beet plot	Baden-Wuerttemberg	Ditzingen Hirschlanden Treschklingen Biberach	48°49′38.1″ N 9°00′47.4″ E 48°49′29.9″ N 9°01′06.0″ E 49°13′17.1″ N 9°03′32.3″ E 49°11′47.5″ N 9°07′03.6″ E	20 20 20 20
	Bavaria	Seligenstadt Giebelstadt Ochsenfurt Biebelsried Gelchsheim Ingolstadt	49°50′27.5″ N 10°05′16.2″ E 49°39′48.2″ N 9°56′26.8″ E 49°37′16.2″ N 10°05′42.8″ E 49°46′36.4″ N 10°04′34.9″ E 49°33′40.0″ N 10°00′41.2″ E 48°42′53.1″ N 11°23′12.9″ E 48°42′40.7″ N 11°22′16.4″ E	20 20 16 20 20 20 20
	Saxony	Arzberg	51°30′32.9″ N 13°09′23.2″ E	20
	Saxony-Anhalt	Kemberg	51°47′47.0″ N 12°39′22.5″ E	20
	Rhineland-Palatinate	Worms-Eich	49°44′53.6″ N 8°25′42.2″ E	40
Red beet plot	Baden-Wuerttemberg	Ditzingen	48°50′21.0″ N 9°00′47.7″ E	24

**Table 5:** Overview of the primer pairs AsecF/AsecR and PsecF/PsecR to detect 16SrXII-A and 16SrXII-P phytoplasmas.

Primer	Sequence (5′ to 3′)	Primer Length (nt)	Amplicon Length (bp)	Reference
AsecF	GGATTGATAGATGCTGCCCCA	21	549	Modified from [62]
AsecR	TGGACTTTGATTGCGAGCGT	20		This study
PsecF	GTCCATAAGGTAAGGAGTAGTAACA	25	857	This study
PsecR	GGAAAGATCAAGGTCAAATCGGTAA	25		This study

## Data Availability

Representative partial *secY* sequences obtained from sugar beet, red beet, and *P. leporinus* samples have been deposited in the NCBI GenBank database under the accession numbers PZ581736–PZ581747.

## Supplementary Materials

The following supporting information can be downloaded at: https://www.mdpi.com/article/10.3390/plants15182823/s1, Figure S1: Comparison of phytoplasma screening results obtained with P1/P7 (A) and the *secY*-based differentiation assays with AsecF/AsecR and PsecF/PsecR (B) using samples from a severely SBR-affected sugar beet plot in Eppingen, Germany. White boxed regions indicate corresponding samples that were analyzed on a separate gel and assembled for presentation. NTC, non-template control (nuclease-free water); PC, positive control (for AsecF/R, DNA from the 16SrXII-A reference strain STOL obtained from C. roseus; for PsecF/R, P1/P7 and R16F2n/R16R2, DNA from a sugar beet sample that tested positive for 16SrXII-P); L, DNA ladder used for fragment size estimation ranging from 2 kb to 50 bp. Figure S2: Comparison of phytoplasma screening results obtained with P1/P7 (A) and the *secY*-based differentiation assays AsecF/AsecR and PsecF/PsecR (B) using samples from a severely RTD-affected sugar beet plots from Jedenspeigen (white numbers) and Ginzersdorf (yellow numbers), Austria. NTC, non-template control (nuclease-free water); PC, positive control (for AsecF/R, DNA from the 16SrXII-A reference strain STOL obtained from *C. roseus*; for PsecF/R and P1/P7, DNA from a sugar beet sample that tested positive for 16SrXII-P); L, DNA ladder used for fragment size estimation ranging from 2 kb to 50 bp. Figure S3: Comparison of phytoplasma screening results obtained with P1/P7 (A) and R16F2n/R16R2 (B), as well as the SecY-based differentiation assays AsecF/AsecR (C) and PsecF/PsecR (D) using samples from a severely affected red beet plot from Ditzingen (Germany). NTC, non-template control (nuclease-free water); PC, positive control (for AsecF/R, DNA from the 16SrXII-A reference strain STOL obtained from *C. roseus*; for PsecF/R, P1/P7 and R16F2n/R16R2, DNA from a sugar beet sample that tested positive for 16SrXII-P); L, DNA ladder used for fragment size estimation ranging from 2 kb to 50 bp. Table S1: Concordances of phytoplasma detections by AsecF/R, PsecF/R, and P1/P7 Assays in the sugar- and red beet samples. Table S2: Concordances of phytoplasma detections by AsecF/R, PsecF/R, and P1/P7 Assays in the planthopper samples. Table S3: Nucleotide sequences of the utilized gBlocks used for detection limit assessment. Table S4: Overview of nucleotide sequences and references used for phylogenetic assignment.
