# Supplementary material for: A Discriminative PCR Assay Enabling Differentiation of ‘Candidatus Phytoplasma solani’ Subgroups 16SrXII-A and -P
Source: Plants (Basel). 2026 Sep 14;15(18):2823. doi: 10.3390/plants15182823 (PMC13610290; doi:10.3390/plants15182823)
Supplement: Supplementary file 1 [file plants-15-02823-s001.zip › plants-4518694-supplementary.pdf]

Supplementary Material

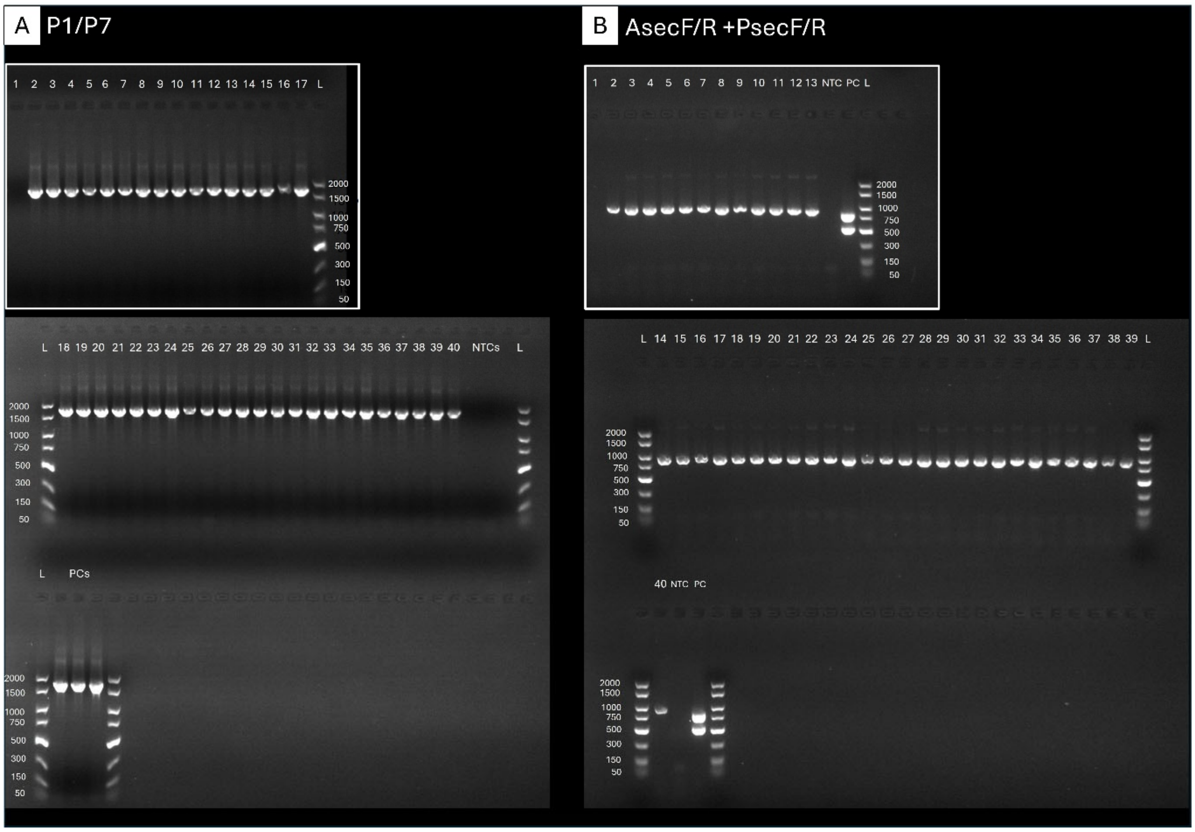

Figure S1. Comparison of phytoplasma screening results obtained with P1/P7 (A) and the *secY*-based differentiation assays with AsecF/AsecR and PsecF/PsecR (B) using samples from a severely SBR-affected sugar beet plot in Eppingen, Germany. White boxed regions indicate corresponding samples that were analyzed on a separate gel and assembled for presentation. NTC, non-template control (nuclease-free water); PC, positive control (for AsecF/R, DNA from the 16SrXII-A reference strain STOL obtained from *C. roseus*; for PsecF/R, P1/P7 and R16F2n/R16R2, DNA from a sugar beet sample that tested positive for 16SrXII-P); L, DNA ladder used for fragment size estimation ranging from 2 kb to 50 bp.

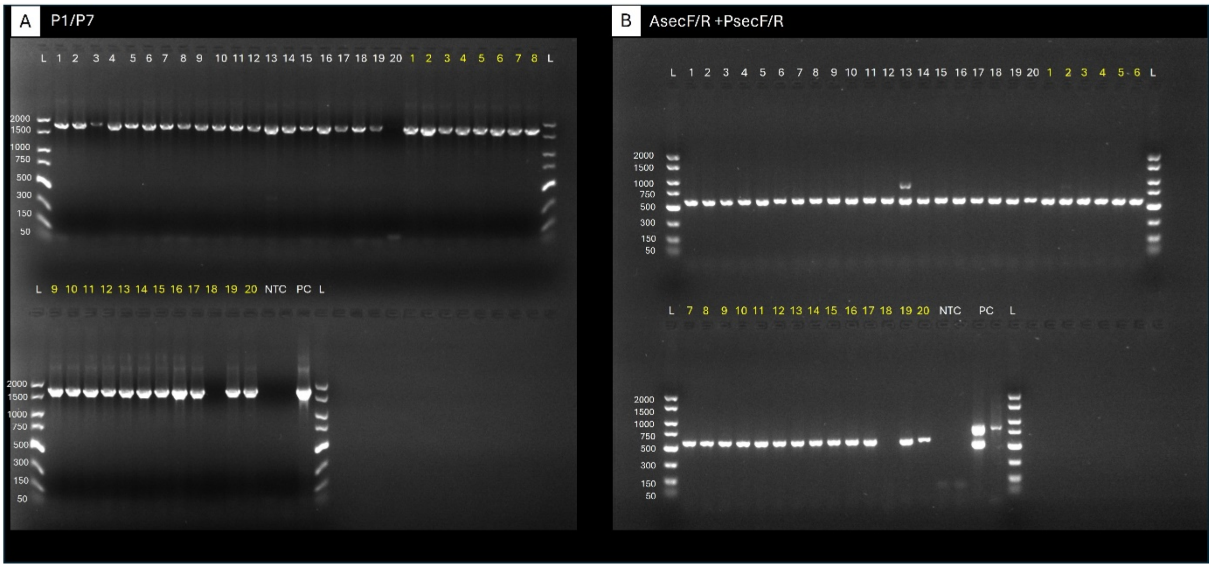

Figure S2. Comparison of phytoplasma screening results obtained with P1/P7 (A) and the *secY*-based differentiation assays AsecF/AsecR and PsecF/PsecR (B) using samples from a severely RTD-affected sugar beet plots from Jedenspeigen (white numbers) and Ginzersdorf (yellow numbers), Austria. NTC, non-template control (nuclease-free water); PC, positive control (for AsecF/R, DNA from the 16SrXII-A reference strain STOL obtained from *C. roseus*; for PsecF/R and P1/P7, DNA from a sugar beet sample that tested positive for 16SrXII-P); L, DNA ladder used for fragment size estimation ranging from 2 kb to 50 bp.

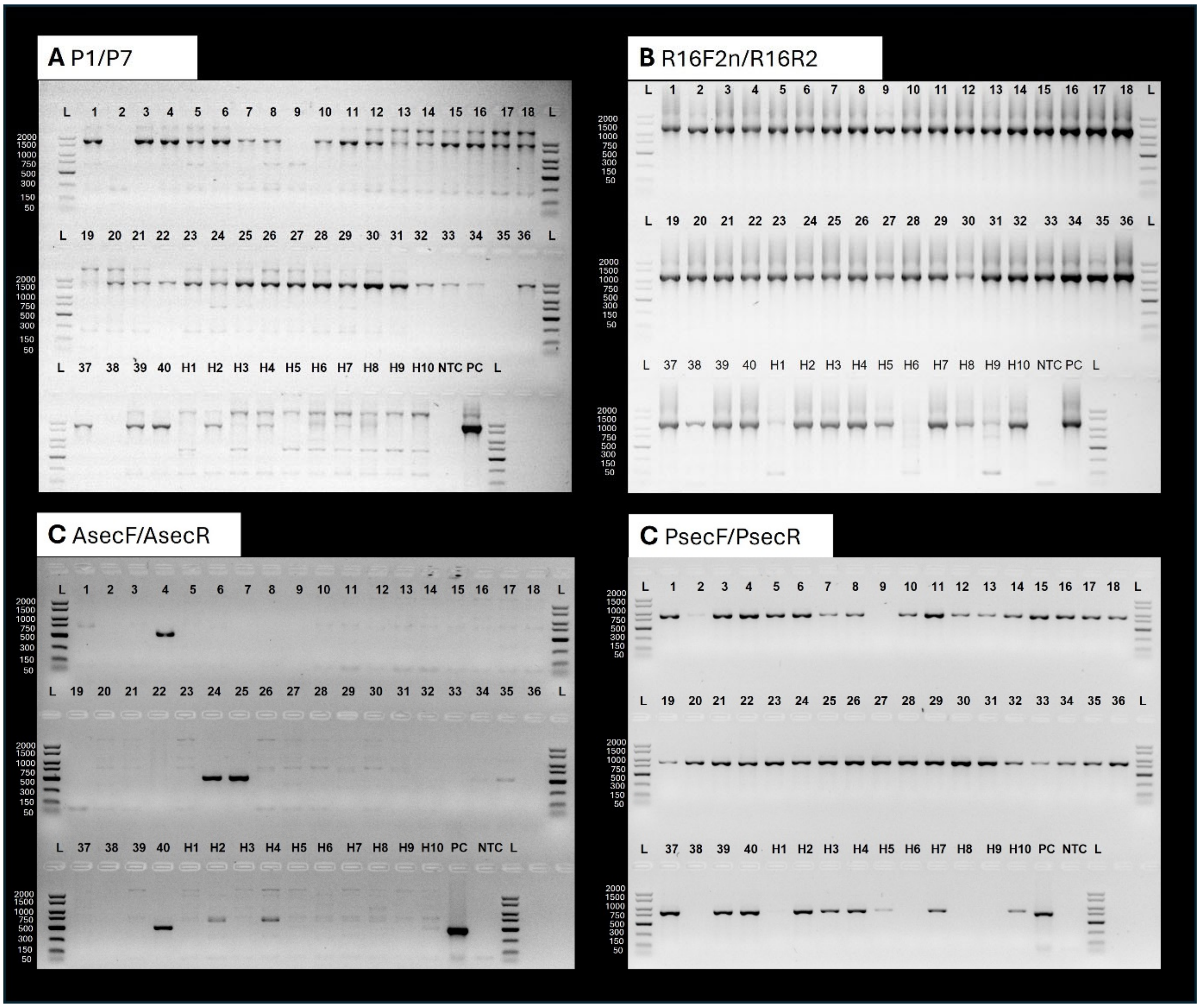

Figure S3. Comparison of phytoplasma screening results obtained with P1/P7 (A) and R16F2n/R16R2 (B), as well as the *secY*-based differentiation assays AsecF/AsecR (C) and PsecF/PsecR (D) using samples from a severely affected red beet plot from Ditzingen (Germany). NTC, non-template control (nuclease-free water); PC, positive control (for AsecF/R, DNA from the 16SrXII-A reference strain STOL obtained from *C. roseus*; for PsecF/R, P1/P7 and R16F2n/R16R2, DNA from a sugar beet sample that tested positive for 16SrXII-P); L, DNA ladder used for fragment size estimation ranging from 2 kb to 50 bp.

Table S1. Concordances of phytoplasma detections by AsecF/R, PsecF/R, and P1/P7 Assays in the sugar- and red beet samples.

Sample source	Country	Federal state/Region	Location	Samples compared (n)	Both positive (n)	Both negative (n)	AsecF/R or PsecF/R positive, P1/P7 negative (n)	AsecF/R and PsecF/R negative, P1/P7 positive (n)	Concordance n (%)
Sugar beet	**Germany**	Baden-Wuerttemberg	Bad Wimpfen	41	26	8	7	0	34 (82.90)
			Hueffenhardt	40	34	5	1	0	39 (97.50)
			Eppingen	40	39	1	0	0	40 (100.00)
			Oberriexingen	40	27	5	8	0	32 (80.00)
			Ditzingen	40	35	2	3	0	37 (92.50)
			Pulverdingen /Markgröningen	34	24	4	6	0	28 (82.35)
		Bavaria	Rodheim	44	43	1	0	0	44 (100.00)
			Rain am Lech /Oberdolling	38	15	20	3	0	35 (92.10)
		Saxony-Anhalt	Gehrden	45	35	9	1	0	44 (97.80)
			Hobeck	40	29	6	3	2	35 (87.50)
			Gommern-Nedlitz	41	11	30	0	0	41 (100.00)
			Vockerode	40	27	9	3	1	36 (90.00)
			Buhlendorf	40	12	27	1	0	39 (97.50)
		Brandenburg	Neureetz	40	34	5	1	0	39 (97.50)
		Rhineland-Palatinate	Worms-Eich	41	28	13	0	0	41 (100.00)
			Bischheim	35	13	22	0	0	35 (100.00)
		North Rhine-Westphalia	Bornheim	41	0	41	0	0	41 (100.00)
			Vettweiß	40	0	40	0	0	40 (100.00)
		**Total**		**720**	**432**	**248**	**37**	**3**	**680 (94.44)**
Red beet		Baden-Wuertemberg	Ditzingen	**50**	**43**	**2**	**1**	**4**	**45 (90.00)**
Sugar beet	France	Great East	Richtolsheim	20	0	20	0	0	20 (100.00)
	Austria	Lower Austria	Ginzersdorf	20	19	1	0	0	20 (100.00)
			Jedenspeigen	20	19	0	1	0	19 (95.00)
	Slovakia	Nitra	Malé Zálužie	20	1	15	4	0	16 (80.00)
	Poland	Lubusz	Siedlisko-Dębianka	20	4	12	4	0	16 (80.00)
			Piotrowice	20	0	20	0	0	20 (100.00)
	The Netherlands	Drenthe	Orvelte	20	0	20	0	0	20 (100.00)
	Belgium	Wallonia	Verlaine	20	0	20	0	0	20 (100.00)
			Donceel	20	0	20	0	0	20 (100.00)
		**Total**		**180**	**43**	**128**	**9**	**0**	**171 (95.00)**

Table S2. Concordances of phytoplasma detections by AsecF/R, PsecF/R, and P1/P7 Assays in the planthopper samples.

Sample source	Federal state	Location	Samples compared (n)	Both positive (n)	Both negative (n)	AsecF/R or PsecF/R positive, P1/P7 negative (n)	AsecF/R and PsecF/R negative, P1/P7 positive (n)	Concordance n (%)
Sugar beet field	Baden-Wuertemberg	Ditzingen	20	10	9	1	0	19 (95.00)
		Hirschlanden	20	10	5	5	0	15 (75.00)
		Treschklingen	20	15	5	0	0	20 (100.00)
		Biberach	20	10	8	0	2	18 (90.00)
	Bavaria	Seligenstadt	20	13	2	1	4	15 (75.00)
		Giebelstadt	20	14	4	2	0	18 (90.00)
		Ochsenfurt	16	6	5	0	5	11 (68.75)
		Biebelsried	20	10	4	1	5	14 (70.00)
		Gelchsheim	20	20	0	0	0	20 (100.00)
		Ingolstadt	20	1	19	0	0	20 (100.00)
			20	4	16	0	0	20 (100.00)
	Saxony	Arzberg	20	18	1	0	1	19 (95.00)
	Saxony-Anhalt	Kemberg	20	0	20	0	0	20 (100.00)
	Rhineland-Palatinate	Worms-Eich	40	17	22	1	0	39 (97.50)
	**Total**		**296**	**148**	**120**	**11**	**17**	**268 (90.54)**
Red beet field	Baden-Wuertemberg	Ditzingen	**24**	**14**	**4**	**0**	**6**	**18 (75.00)**

Table S3. Nucleotide sequences of the utilized gBlocks used for detection limit assessment.

gBlock	Sequence (5' to 3')
AsecF/R	GATCCATGCTAGTCGTACGATTCGACGTCAGGATTGATAGATGCTGCCCCATCAAAACTTTTTGGTTTAGGCATTTATC<br>CTTATATTACAGCTTCTATTGTAGTTCAATTTTTACAAAAACTTTTACCTATTTGCCGTGAGTGGAAAGAACAAGGCCA<br>AATCGGTAAACGTAAACTTAACCTTTTAACTAGGGCTTTAGCTTTATTATTTGTTTTTGGACAAACTTTTGGAATGATTC<br>AAAAAACAAGTGATTCATTGGCAGTTTGCTTTTTGATCCCTTTAATTGCCGCTGCTGGATGTGCTATTTTAATTTGGTTT<br>GCTGATTTAATCAACTCTCAAGGGATTGGTAATGGAACTTCTATTTTAATTATGGCCTCAATGAGTAATAATTTAATTG<br>ACTCTTTAAAAGAAATAAAACAAAATTATTACGACAATTTATTTACCAACAATTTTGACCCAAAATTGCTAACACAAT<br>TTATTTTAATTATCTTAGTATTGCTTTTATTTTTAATTGTAACTGTGATTGTCCAAATTACTTCTTTAAAAATACCAGTAC<br>AATACGCTCGCAATCAAAGTCCACTAGTGACGTTACGTCAGTACGATCGTCGA
PsecF/R	AAGTGTTTGCCGCTTCGGAGTAGCGTCGTAGCTTTGTGTACTGTCAGCATGTCCATAAGGTAAGGAGTAGTAACAAGA<br>GTAATCATAAAAAGAGTACCTATAACAGTGATTTTAAACAAAAGAGAAGATAAATAATAAGTGGTTTGCTCTCCAGG<br>TCTTAAACCGGCAATATAAGCGTCTTGTTTTGATAAATGTTCGGAAATTTCTTCAGGATTAACATTCATAAAAGTAGAG<br>AAAAATGAAAAAACAATGATTAATAAAATATGTAACCCAAAAGCAAAATTAATAATTTTAATATTAGTTAAATATGT<br>AATAATGTTATTAAATGTTTGATTATTGATTACTTGAGTTAAAAAATTAAAAGGTTGCAACAAGGCATTGGCTAAAAT<br>CAAAGGCATAACTCCAGCAGTGTTAATTTTCAAAGGGATATAACTATTACCTTGAGATGCAACTTGATTGCGAGCATA<br>ATGAATTGGTATTTTCAAAGAAGTTATTTGAACAATCACAGTAATAACTAAAAACAAGATCAAAACTAAAACAATAA<br>TAATAAAATTTATAAACTCTTTTGCATTAAAACTTTGAACCATTGGTAGCAAAAAAATGTTTTTTAATTCTTGAAAAGA<br>AGAAATTAAGCTACCACTCATAGAAGCTATAATCAAAAGAGAAGTACCATTACCAATTCCTTTAGAATTGATTAAATC<br>AGCAAACCAAATGAGAATAGCACATCCCGCTACAGCAATCAAAGAGATTAAAAAGCAATCTCCCGACAAAATTTTTG<br>ATTCTGGTGATAATAATACTTTATTAAAATTATTAAGAAAGGCAAAAGTTTGTCCAAAAACAAATAAAAGAGCTAAA<br>ACGCGAGTTAAAAGATTAAGTTTTCGTTTACCGATTTGACCTTGATCTTTCCAAATGGGTGCAGGACTAATCAGTGCTT<br>TCGTTCGCACGTCGGGTTCTTTG

Table S4. Overview of nucleotide sequences and references used for phylogenetic assignment.

16S Group	Provisional Taxon	Strain / Isolate	Host	GenBank Accession / Reference
16SrXII-A	'*Ca*. P. solani'	c1	Bindweed	CP103788.1
		c4	Bindweed	CP103787.1
		c5	Bindweed	CP103786.1
		o3	Stinging nettle	CP103785.1
		POT	*Hyalesthes+ obsoletus*	CM135798.1
		135-24	*Reptalus artemisiae*	CM148571.1
		P5	Potato	PP270182.1
		P8	Potato	PP270181.1
		A672A	Sugar beet	PZ581737 (This study)
		RB25A	Red beet	PZ581740 (This study)
		RB40A	Red beet	PZ581736 (This study)
		GEL9A	*Pentastiridius leporinus* from sugar beet	PZ581738 (This study)
		GEL19A	*P. leporinus* from sugar beet	PZ581739 (This study)
		SS15	*P. leporinus* from sugar beet	PZ581741 (This study)
16SrXII-B/-C	'*Ca*. P. australiense'	PAa	Cotton	AM422018.1
		NZSb11	Strawberry	CP002548.1
16SrXII-D	'*Ca*. P. japonicum'	JHP	*Hydrangea macrophylla*	AB738739.1
16SrXII-E	'*Ca*. P. fragariae'	CN-148	Potato	KJ144894.1
		YN-10G	Potato	KJ144895.1
16SrXII-I		YN-169	Potato	KJ144896.1
16SrXII-P	'*Ca*. P. solani'-related phytoplasma	GOE	*P. leporinus*	CP155828.1
		PENLEP	*P. leporinus*	Vogel et al. 2025
		GEL9P	*P. leporinus* from sugar beet	PZ581746 (This study)
		GEL18P	*P. leporinus* from sugar beet	PZ581747 (This study
		INS18P	*P. leporinus* from red beet	PZ581742 (This study)
		INS20P	*P. leporinus* from red beet	(This study)
		916/22	Sugar beet	OQ717010.1
		PL359/23	Sugar beet	PP731993.1
		SB-215	Sugar beet	PP270180.1
		A134P	Sugar beet	PZ581744 (This study)
		A672P	Sugar beet	PZ581745 (This study)
		RB30P	Red beet	PZ581743 (This study)
		RB37P	Red beet	(This study)
16SrI-A	'*Ca*. P. asteris'	M33	Carrot	CP128397.1
